# Intrinsic functional networks for distinct sources of error in visual working memory

**DOI:** 10.1093/cercor/bhae401

**Published:** 2024-10-09

**Authors:** Xuqian Li, Lena K L Oestreich, Dragan Rangelov, Delphine Lévy-Bencheton, Michael J O’Sullivan

**Affiliations:** Institute for Molecular Bioscience, The University of Queensland, 306 Carmody Road, St Lucia QLD 4067, Australia; Australian Institute for Bioengineering and Nanotechnology, The University of Queensland, Corner College Road and Cooper Road, St Lucia QLD 4067, Australia; Australian Institute for Bioengineering and Nanotechnology, The University of Queensland, Corner College Road and Cooper Road, St Lucia QLD 4067, Australia; School of Psychology, The University of Queensland, Sir Fred Schonell Drive, St Lucia QLD 4067, Australia; National Imaging Facility, The University of Queensland, University Drive, St Lucia QLD 4067, Australia; Queensland Brain Institute, The University of Queensland, QBI Building 79, St Lucia QLD 4067, Australia; School of Economics, The University of Queensland, 39 Blair Drive, St Lucia QLD 4067, Australia; Mater Research, Raymond Terrace, South Brisbane QLD 4101, Australia; Institute for Molecular Bioscience, The University of Queensland, 306 Carmody Road, St Lucia QLD 4067, Australia; Department of Neurology, Royal Brisbane and Women’s Hospital, Butterfield Street, Herston QLD 4006, Australia

**Keywords:** brain-behavior associations, delayed estimation task, mixture distribution modeling, resting-state networks, visual working memory

## Abstract

Visual working memory (VWM) is a core cognitive function wherein visual information is stored and manipulated over short periods. Response errors in VWM tasks arise from the imprecise memory of target items, swaps between targets and nontargets, and random guesses. However, it remains unclear whether these types of errors are underpinned by distinct neural networks. To answer this question, we recruited 80 healthy adults to perform delayed estimation tasks and acquired their resting-state functional magnetic resonance imaging scans. The tasks required participants to reproduce the memorized visual feature along continuous scales, which, combined with mixture distribution modeling, allowed us to estimate the measures of memory precision, swap errors, and random guesses. Intrinsic functional connectivity within and between different networks, identified using a hierarchical clustering approach, was estimated for each participant. Our analyses revealed that higher memory precision was associated with increased connectivity within a frontal-opercular network, as well as between the dorsal attention network and an angular-gyrus-cerebellar network. We also found that coupling between the frontoparietal control network and the cingulo-opercular network contributes to both memory precision and random guesses. Our findings demonstrate that distinct sources of variability in VWM performance are underpinned by different yet partially overlapping intrinsic functional networks.

## Introduction

Visual working memory (VWM) refers to the ability to hold and manipulate visual information over short periods of time. Since the pioneering work by Bays et al. (2009), previous studies have sought to understand variability in VWM performance through the segregation of different sources of error. Hypothetically, internal representations stored in working memory reflect an imprecise representation of a target item, sometimes along with representations of nontarget items. These representations contain information about the spatial location and visual attributes of the encoded items (Logie 1986, 1995). Response errors can arise from imprecision in these representations, thought to originate from the inherent noise within the working memory system. Alternatively, errors can arise from erroneous binding between different features of the target and nontarget items (e.g. binding location of the target item with the orientation of a nontarget item), so-called “swap errors” (Bays et al. 2009). Finally, response errors can arise from random guessing when sensory encoding or maintenance of the encoded information fails (Bays et al. 2009). This influential framework challenges the long-standing model of VWM, which holds that a fixed number of memory “slots” are used to store a fixed number of visual items (Zhang and Luck 2008). Instead, Bays et al. framework has led to the development of resource-based models of VWM, which suggest that a single memory resource is distributed among visual items. As the number of items increases, the representation of each individual item becomes less precise, resulting in more frequent swaps and guesses (Bays et al. 2009; Ma et al. 2014). The framework has also enabled researchers to gain a deeper understanding of individual differences in VWM performance. For example, among children aged 7 to 13 yrs, improvements in overall task performance were primarily driven by enhanced age-related memory precision rather than by decreases in swap errors or random guesses (Burnett Heyes et al. 2012; Burnett Heyes et al. 2016).

Despite the theoretical and practical implications of the mixture component framework, only a few studies to date have examined the neural correlates of the distinct sources of variability in VWM performance. Lugtmeijer et al. (2021), for example, investigated feature recall and binding in stroke patients and found that deficits in memory precision were associated with lesions in the left superior parietal lobule and auditory cortex and the right inferior frontal gyrus and supplementary motor area, whereas deficits in feature binding were specifically associated with lesions in the left postcentral gyrus (Lugtmeijer et al. 2021). By examining individual differences in white matter microstructural properties among healthy adults, our recent work revealed associations between sources of error in VWM tasks and long-range association fibers (Li et al. 2023). We found that memory precision was specifically associated with fiber coherence of the bilateral superior longitudinal fasciculus and inferior fronto-occipital fasciculus, whereas random guessing was specifically related to axonal density of the inferior fronto-occipital fasciculus. Of note, the associations we found were generalized across 2 working memory tasks examining different visual features: one spatial task, where participants recalled the location of a target item, and one object task, where they recalled the orientation of the target item.

The previous findings suggest that the distinct sources of error in VWM performance are supported by distinct white matter tracts and cortical regions that work in unison to enable veridical memory storage and retrieval. This raises the question of whether these errors are also supported by different patterns of spontaneous functional brain activity. A significant aspect of the spontaneous brain activity is its ability to show reproducible temporal correlations among brain regions, thus revealing intrinsic networks of the brain (Fox et al. 2005; Vincent et al. 2008; Yeo et al. 2011). The topographical properties, along with the internal and external dynamics of these networks, can be analyzed to understand brain activity over timescales from ten to hundreds of seconds. Moreover, the macroscale organization reflected by these intrinsic networks often closely mirrors the functional networks engaged during a wide range of tasks, providing insights beyond what structural imaging alone can offer.

Studies employing resting-state functional magnetic resonance imaging (rs-fMRI) have revealed links between VWM performance and functional connectivity (FC) within and between several intrinsic brain networks. For example, intrinsic FC within the dorsal attention network, a network crucial for top-down attention (Corbetta and Shulman 2002; Vossel et al. 2014), was associated with working memory accuracy (Ren et al. 2019). Additionally, training-induced changes in FC within the frontoparietal control network, a network for executive control and adaptive behavior (Vincent et al. 2008; Dixon et al. 2018), were correlated with improved task accuracy (Jolles et al. 2013). Moreover, individual differences in the extent to which the default mode network, typically deactivated during cognitively demanding tasks (Fox et al. 2005; Yeo et al. 2011), and the frontoparietal control network were anticorrelated at resting state were associated with individual differences in working memory capacity (Sala-Llonch et al. 2012; Keller et al. 2015). Taken together, prior studies have shown relationships between aggregate VWM performance and several intrinsic functional networks. Importantly, however, they have overlooked the heterogeneous nature of VWM performance and have not distinguished between memory precision, binding failures, and random guessing. Whether and how distinct sources of errors in task performance are related to intrinsic functional networks remains unclear.

In this study, we sought to characterize the relationships between distinct sources of behavioral variability in VWM and intrinsic functional networks. To achieve this goal, we acquired rs-fMRI and computed FC between 37 regions of interest (ROIs). A data-driven hierarchical clustering approach was applied to reveal different functional networks, followed by calculating the average strength of FC within and between different networks. To evaluate VWM performance, we used a delayed estimation task, as employed in our previous work (Li et al. 2023), which required participants to encode visual gratings that varied in both their spatial location and orientation. Following a short delay period, participants reported on a continuous scale either the orientation or location of only one of the gratings. The location and orientation tasks were used to investigate the effects of spatial and nonspatial visual features on relationships between functional brain networks and measures of working memory performance. Estimated measures of memory precision, swap errors, and random guesses were obtained by modeling different sources of error using computational modeling (Bays et al. 2009).

Building on our previous work (Li et al. 2023) and findings from Lugtmeijer et al. (2021), which suggest the existence of specific neural systems for memory precision, swap errors, and random guesses, we hypothesized that distinct VWM errors would be associated with distinct intrinsic functional networks. Specifically, for memory precision, we considered the frontoparietal control network and dorsal attention network as the prime candidates, due to their spatial correspondence with the superior longitudinal fasciculus (Vincent et al. 2008; Vossel et al. 2014; Li et al. 2023). Furthermore, we expected swap errors, previously linked to lesions in the postcentral gyrus (Lugtmeijer et al. 2021), to be associated with the somatomotor network. Additionally, we hypothesized that random guesses, often stemming from attentional lapses, mind-wandering, and fatigue, would be linked to the default mode network, which is crucial for regulating internally directed cognition (Buckner et al. 2008; Christoff et al. 2016). Since our prior study showed that the observed associations were generalized across working memory tasks examining spatial and nonspatial visual features (Li et al. 2023), we predicted in this study that the relationships between intrinsic networks and different error types would not vary specifically between the location and orientation tasks.

## Materials and methods

### Participants

Eighty-seven healthy adult humans were recruited from the University of Queensland through an online volunteer system. Participants underwent a behavioral session to perform the VWM experiment, followed by an MRI. Seven participants were excluded due to either data corruption (*n* = 4) or incomplete brain imaging data acquisition (*n* = 3). The final sample included 80 participants aged 18 to 38 yrs (*M* = 24.24, *SD* = 4.61; 39 females). All participants completed safety screening questionnaires and provided written informed consent before the experimental sessions. Participants were reimbursed at a rate of $20 per h. The study was approved by the Human Research Ethics Committee of The University of Queensland (2018001427).

### Experimental procedure

**Fig. 1:**
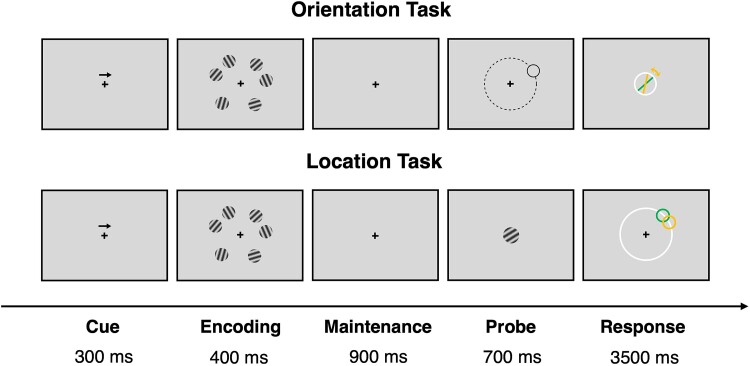
Schematic illustration of the delayed estimation tasks. At the beginning of each trial, an arrow cue appeared to remind participants to encode items presented on either the left or right side of the screen. Six differently oriented gratings were then presented during the encoding period, followed by a maintenance period. In the *Orientation Task*, the location of one of the memorized items was presented as a probe to indicate which item would be retrieved. During the response period, participants reported the orientation of the target item. In the *Location Task*, the orientation of one of the memorized items was presented as a probe and participants reported the location of the target item during the response period.

The delayed estimation experiment has been described in detail in our previous study (Li et al. 2023). Briefly, the experiment was implemented in MATLAB R2018a (MathWorks, Natick, MA) using Psychtoolbox (Brainard 1997; Pelli 1997). Of note, the experiment was performed concurrently with electroencephalography recording, but these data are not reported here. Each trial started with the presentation of a central arrow cue pointing to either left or right (Fig. 1). This manipulation was designed to direct participants’ attention to the task-relevant visual hemifield. The cue was followed by an encoding phase for 400 ms, during which 6 differently oriented gratings were presented simultaneously and bilaterally (i.e. 3 in each visual hemifield). Based on the direction of the cue arrow, participants had to focus only on the 3 gratings presented on the left or right hemifield. The encoding phase was followed by a 900 ms maintenance phase, during which only the central cross remained on the screen. Next, a response probe appeared, showing either the location or orientation of one of the memorized gratings for 700 ms. In the orientation task, a probe stimulus was displayed at the location of a randomly chosen grating. As the probe disappeared, participants were instructed to report the orientation of the target item in a white circle. In the location task, by contrast, the probe indicated the orientation of a randomly chosen grating. As the probe disappeared, participants were instructed to report the location of the target item on a white circle. For both tasks, participants had maximally 3,500 ms to respond. After the response, or at the end of the response period, feedback was provided for 1,000 ms in the form of a green line to show the correct orientation or a green circle to show the correct location.

The experiment consisted of 4 randomized blocks so that each of the tasks was presented twice. Each block contained 2 runs in which participants were cued to encode items on the left side of the screen and 2 runs in which participants were cued to encode items on the right side, and these alternated with each other. The runs were counterbalanced across participants. Each block comprised 120 trials, with 30 trials per run. A total of 480 trials were collected from each participant.

### Behavioral analyses

To quantify behavioral performance in each trial, response errors were computed as the angular difference between the participant’s response and the correct orientation or location of the probed item. In the orientation task, response errors ranged between 0° and ± 90°. In the location task, response errors ranged between 0° and ± 180°, with errors larger than ±90° indicating that participants selected a location in the uncued hemifield. Initial data inspection indicated that participants never made such “hemifield-swap” errors. Thus, in later analyses, item location was treated similarly to item orientation, with the range of response errors being between 0° and ± 90°. Response errors in both tasks were transformed from degrees to pi radians (πrad) with 0° and ± 90° mapped to 0 πrad and ± 1 πrad, respectively. A histogram of response errors was constructed for each participant and each task.

To identify outlier participants, error distributions per task were compared against a uniform distribution (i.e. a uniform distribution is expected if a participant guessed in a majority of trials) for each participant, using the Kolmogorov–Smirnov test (Massey 1951). Participants whose error distributions proved to be uniformly distributed at the level of *P* < 0.05 were removed from further analyses. Based on this criterion, 8 participants were excluded, leaving a total of 72 participants (36 females; 18 to 38 yrs, *M* = 24.31, *SD* = 4.77) for the following analyses.

To identify different sources of behavioral variability in VWM tasks, a mixture distribution modeling introduced by Bays et al. (2009) was applied, which attributes response errors to a mixture of 3 components. Briefly, the model is defined as the probability of reporting the target item (P*_T_*), the probability of reporting the nontarget items (P*_NT_*), the probability of random guessing (P*_G_*), and the concentration parameter κ of the von Mises distribution that described the variability around the target value. The maximum likelihood estimates of the parameters were obtained separately for each participant in each task using an expectation–maximization algorithm. The fitted von Mises κ was converted to circular standard deviation (σ_vM_) as defined by Fisher (1995), giving an inverse measure of *memory precision* that reflects the precision of representations stored in VWM (Bays, Gorgoraptis, et al. 2011a; Bays, Wu, et al. 2011b; Pratte et al. 2017). P*_NT_* measures *swap errors*, which describe the proportion of responses arising from feature binding anomalies in working memory where a nontarget feature is “swapped in” for the target feature (Bays 2016; Schneegans and Bays 2017). P*_G_* measures the *random guesses* which reflect the proportion of responses originating from attention lapses, poor task compliance or other motivational factors. These analyses were performed using the Analogue Report Toolbox in MATLAB R2021b (Bays et al. 2009; Schneegans and Bays 2016). Further analyses were conducted to assess the recoverability of model parameters, which demonstrated that all parameters were readily recoverable (White et al. 2018; Wilson and Collins 2019; see Supplementary Methods).

To explore whether behavioral performance differs in the orientation and location tasks, we conducted paired-samples *t*-tests to examine the differences in σ_vM_, P*_T_*, P*_NT_*, and P*_G_* between tasks. The significance level was set at *P* < 0.05, corrected for multiple comparisons using Bonferroni adjustment. These analyses were performed using R package “rstatix” (Kassambara 2020).

### Neuroimaging analysis

#### Image acquisition

Participants underwent MRI scans using a Siemens Magnetom Prisma 3 T system at the Centre for Advanced Imaging at The University of Queensland. T1-weighted structural scans were obtained with a magnetization-prepared 2 rapid acquisition gradient echo sequence (Marques et al. 2010), with field-of-view = 240 mm, number of slices = 176, TR = 4000 ms, TE = 2.92 ms, TI 1 = 700 ms, TI 2 = 2220 ms, first flip angle = 6°, second flip angle = 7°, and 5 to 6 min of acquisition time. Whole-brain blood-oxygen-level-dependent (BOLD) signals were acquired using an echo-planar imaging sequence with field-of-view = 206 mm, number of slices = 60, slice thickness = 2.4 mm, TR = 820 ms, TE = 33 ms, and flip angle = 53°, and acquisition time of about 7 min. Participants were instructed to close their eyes and relax while remaining awake during the resting-state functional scans.

#### Image Preprocessing and Denoising

Data were preprocessed using the CONN toolbox (v21.a; Nieto-Castanon 2020) and Statistical Parametric Mapping (SPM) 12 (https://www.fil.ion.ucl.ac.uk/spm/software/spm12/). Functional data were corrected for subject movement and magnetic susceptibility geometric distortions (Andersson et al. 2001). Temporal misalignment between slices was corrected using SPM12 slice-timing correction procedure (Henson et al. 1999; Sladky et al. 2011). Potential outlier scans were identified using artifact detection tools as scans with framewise displacement above 0.9 mm or global signal changes above 5 standard deviations (Power et al. 2014). Functional and T1-weighted data were normalized into standard MNI152 space, segmented into gray matter, white matter, and cerebrospinal fluid tissue classes using SPM unified segmentation and normalization algorithm (Ashburner and Friston 2005). BOLD data were spatially smoothed with an 8 mm full-width-at-half-maximum Gaussian kernel.

The BOLD signal timeseries was denoised by regressing out noise components from cerebral white matter and cerebrospinal fluid areas (Behzadi et al. 2007), subject-motion parameters and their first-order derivatives (Friston et al. 1996), and outlier scans (Power et al. 2014). This was followed by bandpass frequency filtering of the BOLD timeseries between 0.008 Hz and 0.1 Hz (Hallquist et al. 2013). The anatomical component-based noise correction within white matter and cerebrospinal fluid was estimated by computing the average BOLD signal as well as the largest principal components orthogonal to the BOLD average, motion parameters, and outlier scans within each participant’s eroded segmentation masks (Behzadi et al. 2007; Chai et al. 2012).

#### First-level analysis

Thirty-seven ROIs were selected on the basis of a recent meta-analysis of task-based fMRI experiments of VWM (Li et al. 2022). This meta-analysis synthesized BOLD activations during the delay period of VWM tasks across 30 experiments involving 515 healthy young adults. The MNI coordinates of each ROI were defined according to the local peak coordinates obtained from the meta-analysis using the seed-based *d* mapping algorithm (Table S1). A spherical ROI with a radius of 4 mm was created for each MNI coordinate (Fig. 3b). FC between each pair of ROIs was measured by the Fisher-transformed Pearson’s cross-correlation coefficient from a weighted general linear model at the subject level (Nieto-Castanon 2020).

#### Second-level analysis

In this study, we identified different intrinsic functional networks using complete-linkage clustering, a data-driven agglomerative hierarchical clustering procedure (Sorensen 1948; Nieto-Castanon 2020). Hierarchical clustering has been used to analyze rs-fMRI data in many influential studies (e.g. Cordes et al. 2002; Salvador et al. 2005), proving to be as valid as other multivariate approaches such as independent component analysis (Wang and Li 2013). This method stratifies data into hierarchical structures (Zhou et al. 2006; Boly et al. 2012), which aligns conceptually with our understanding of neural networks related to working memory. Although the ROIs we chose were co-activated during VWM tasks and could appear to constitute a single large network, we believed that multiple functionally dissimilar subnetworks were embedded within to support working memory. Therefore, we expected that some well-established intrinsic functional networks, consistently revealed by other approaches (Damoiseaux et al. 2006; Seeley et al. 2007), would be identified through hierarchical clustering. Additionally, using a data-driven approach instead of focusing on predefined networks enables us to study networks that were previously unidentified in research.

The clustering process starts with each region as its own cluster. In each iteration, the pairwise distance between all regions is calculated, and the maximum distance between any 2 regions, each belonging to one of the clusters, is defined as the distance between 2 clusters. The 2 clusters with the shortest distance among all cluster pairs are joined together. This process continues until the optimal number of clusters is reached, which is determined by a point in the hierarchy that corresponds to a jump in the linkage distance. Mathematically, the distance between clusters can be described by the following expression:


$$ D\left(X,Y\right)=\max \left\{d\left(p,q\right)|p\in X,q\in Y\right\} $$

where $X$ and $Y$ denote 2 clusters of regions. $d\left(p,q\right)$ is the distance between region $p\in X$ and $q\in Y$.

The distance between regions $p$ and $q$ is defined as the weighted sum of the *functional similarity metric*, given by the squared Euclidean distance between the group-average FC patterns of the regions, and the *anatomical proximity metric*, given by the squared Euclidean distance between the centroid coordinates of the regions. Formally, $d\left(p,q\right)$ is described as follows:


$$ d\left(p,q\right)=r\times \sum_{j=1}^m{\left({\overline{F}}_{pj}-{\overline{F}}_{qj}\right)}^2+\left(1-r\right)\times \sum_{k=1}^3{\left({C}_{pk}-{C}_{qk}\right)}^2 $$

where ${\overline{F}}_{pj}$and ${\overline{F}}_{qj}$ are the group-average connectivity coefficients of regions $p$ and $q$ with respect to the $j$-th region of the $m$ ROIs. ${C}_{pk}$ and ${C}_{qk}$ refer to the $k$-th coordinates of the centroids of regions $p$ and $q$. $r$ is the weighting parameter, ranging from 0 to 1. For the following analysis, a default weighting parameter $r$ = 0.95 was set to allow a strong contribution of functional similarity between regions while considering a small contribution of anatomical proximity (Nieto-Castanon 2020).

Multivariate analyses were conducted to test whether the FC of the identified clusters differed significantly from zero, using Functional Network Connectivity parametric statistics (Jafri et al. 2008). For each connection between pairs of ROIs, a separate general linear model was defined, with the bivariate correlation coefficient at this connection as the dependent variable. Hypothesis testing at the connection level was performed with random effects across participants and sample covariance estimation across multiple measurements. Statistical inference was performed at the level of individual clusters based on the multivariate parametric statistics within and between each pair of clusters (Jafri et al. 2008), with a cluster-level threshold at *P* < 0.05, corrected for false discovery rate (FDR; Benjamini and Hochberg 1995), together with a post hoc connection-level threshold at uncorrected *P* < 0.05 to identify individual connections showing some of the largest effects within each significant cluster. For each participant, we calculated the average FC within and between the identified clusters, including all related ROIs, regardless of whether individual connections were above the threshold or not. These measures of *within-network FC* and *between-network FC* were then used for subsequent analysis of brain-behavior association.

### Brain-behavior association analysis

To examine the relationship between intrinsic functional networks and distinct sources of error in task performance, we applied linear mixed-effects models to regress memory precision, swap errors, and random guesses separately against FC within and between the identified networks, using R packages “lme4” (Bates et al. 2015) and “lmerTest” (Kuznetsova et al. 2017). To find the most parsimonious model that provided the best fit to the data, a step-up model-building approach was used. This procedure starts with the construction of a base model, followed by the stepwise addition of predictor variables. The base model comprised a fixed intercept, a fixed effect of task (location vs. orientation), and a random intercept of subject to account for the within-subject design. The fixed effects of within- and between-network FC and their interactions with the task effect were tested. All models were ordered based on their restricted log-likelihood obtained via restricted maximum likelihood estimation. Every new model was evaluated against its nested model via the likelihood ratio test, with models refitted by the unrestricted maximum likelihood estimation. In the case of significant interactions, the corresponding main effect terms were also retained. Significant interactions were followed up using simple slope analysis as implemented in the “interactions” package (Long 2022). The significance level was set at *P* < 0.05; *P*-values for fixed effects were calculated using Satterthwaite approximations. Parametric bootstrapping was performed to compute confidence intervals for the parameters of the best-fitting models using the percentile method with 10,000 simulations. Additional analyses of influential cases were conducted using the “influence.ME” package (Nieuwenhuis et al. 2012).

## Results

### Behavioral results

**Fig. 2:**
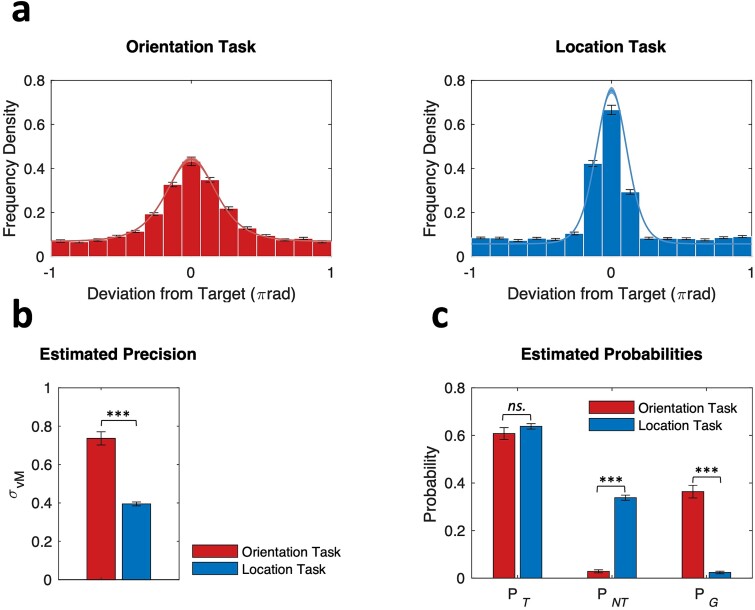
Response error distributions and mixture distribution model fits. (a) Histograms of response deviation relative to the target value for both tasks. Solid lines show the mean of model fits across participants, with shading areas showing ±1 SEM. (b) The estimates of memory precision as measured by the circular standard deviation of von Mises κ and (c) probabilities of reporting the target and nontarget items, and random guessing across participants for both tasks. Error bars denote ±1 SEM.

Figure 2a shows the empirical response error distributions for both orientation and location tasks, and the corresponding model fits predicted by the mixture distribution model. The presence of nonuniform, bell-shaped distributions centered on zero suggests that participants were successful in reporting features of the target item in most trials. The distribution of response errors was wider for the orientation task compared to the location task, indicating higher variability in the former. The mixture distribution model provided good fits to the empirical data for both tasks (Fig. 2a), with the estimated σ_vM_ being significantly higher for the orientation task (*M* = 0.74, *SEM* = 0.03) than for the location task (*M* = 0.40, *SEM* = 0.01) as shown in Fig. 2b, *t*(71) = 10.40, *P* = 6.28e-16. The error distributions for both tasks also displayed long tails, indicating the presence of swap errors and random guesses. Figure 2c shows the differences in the model-estimated probabilities between tasks. The estimated probabilities of target response, measured by the P*_T_*, were similar in the orientation (*M* = 0.61, *SEM* = 0.02) and location tasks (*M* = 0.64, *SEM* = 0.01), *t*(71) = −1.41, *P* = 0.652. The occurrence of swap errors, measured by the P*_NT_*, was significantly lower in the orientation task (*M* = 0.03, *SEM* = 0.01) than in the location task (*M* = 0.34, *SEM* = 0.01), *t*(71) = −28.10, *P* = 3.71e-40. The occurrence of random guessing, measured by the P*_G_*, was significantly higher in the orientation task (*M* = 0.36, *SEM* = 0.03) than in the location task (*M* = 0.02, *SEM* = 0.01), *t*(71) = 13.20, *P* = 1.02e-20.

### Identification of intrinsic functional networks

**Figure 3:**
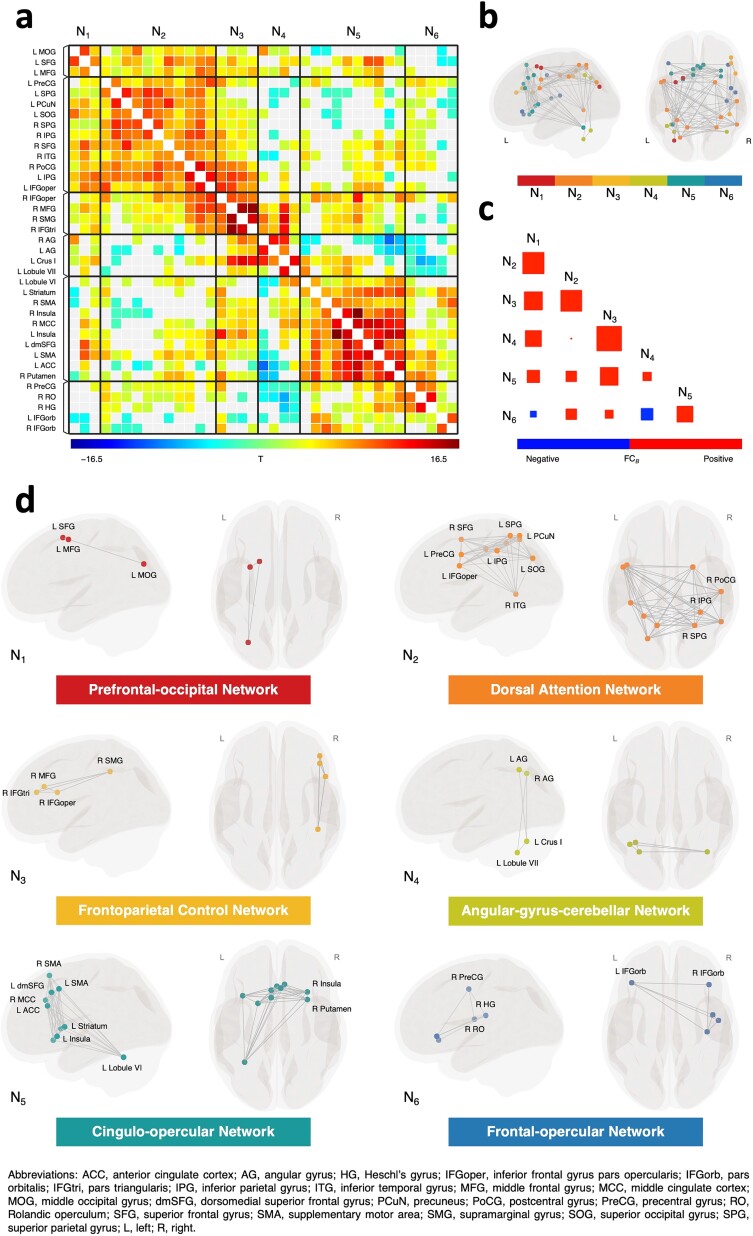
Intrinsic functional networks identified by hierarchical clustering. (a) FC matrix showing a pattern of suprathreshold FC within and between each cluster of ROIs. Rectangles with solid black lines denote significant clusters of connections, with FDR-corrected cluster-level *P* < 0.05. Nonsignificant connections with a post hoc connection-level *P* > 0.05 are shown in light gray. (b) Spherical ROIs included in the hierarchical clustering analysis are shown in MNI space, color-coded based on their network membership following the clustering analysis. (c) Correlation plot showing the between-network FC. ROI-to-ROI connections for each network pair are averaged across participants, with larger size of squares denoting stronger correlation. (d) Identified intrinsic functional networks visualized in glass brains, with ROIs presented in MNI space.

Using the data-driven hierarchical clustering approach, we identified 6 intrinsic functional networks (Fig. 3a, b, and d; Table S1). Network 1 (N_1_) is specific to the left hemisphere, and it comprises 2 prefrontal ROIs in the superior and middle frontal gyrus and the middle occipital gyrus. Network 2 (N_2_) is the largest network identified by the clustering procedure, which corresponded well to the dorsal attention network responsible for top-down attention (Corbetta and Shulman 2002; Vossel et al. 2014). It comprised the bilateral intraparietal sulcus, the left inferior frontal junction and precuneus, and the right human frontal eye field and inferior temporal gyrus. Network 3 (N_3_) comprised 4 regions commonly found in the frontoparietal control network (Vincent et al. 2008; Dixon et al. 2018), including the right middle frontal gyrus, the pars triangularis and pars opercularis of the inferior frontal gyrus, and the right supramarginal gyrus. Network 4 (N_4_) comprised the bilateral angular gyrus and the Crus I and lobule VII in the left posterior cerebellum. Network 5 (N_5_), the second largest network, showed some overlap with the cingulo-opercular network (Seeley et al. 2007; Han et al. 2018). It comprised the bilateral supplementary motor areas, anterior insula, dorsomedial superior frontal gyrus, anterior and mid-cingulate cortices, dorsal striatum, as well as the left cerebellar lobule VI. Finally, network 6 (N_6_) comprised the pars orbitalis of the bilateral inferior frontal gyrus and the right precentral gyrus, Rolandic operculum, and Heschl’s gyrus.

The FC between all pairs of ROIs within each network, as well as between pairs of ROIs in any 2 networks, was significantly different from zero at the cluster level, as demonstrated by the multivariate parametric analyses (Fig. 3a). Next, for each participant, we calculated the average FC within and between the identified networks for subsequent analysis of brain-behavior association (Table S2). Most of the identified networks demonstrated positive correlations with the other networks, except for N_6_, which was negatively correlated with N_1_ and N_4_ (Fig. 3c).

### Brain-behavior associations

**Fig. 4:**
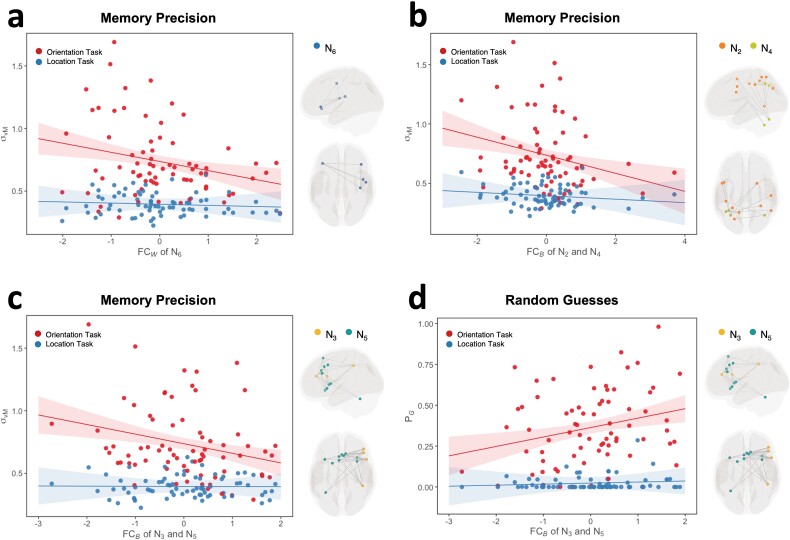
Models of memory precision and random guesses. Effects of (a) within-network FC of N_6_, (b) between-network FC of N_2_ and N_4_, and (c) between-network FC of N_3_ and N_5_ at each level of task on memory precision. (d) Effects of between-network FC of N_3_ and N_5_ at each level of task on random guesses. In all interaction plots, solid lines represent the estimated marginal effects, and shaded areas indicate the 95% confidence intervals. Note: The circular standard deviation of the von Mise concentration parameter σ_vM_ provides an inverse measure of memory precision.

The model comparison and selection processes for all dependent variables are summarized in Tables S3 to S5. In the best-fitting model of memory precision, there was a significant main effect of the within-network FC of N_6_, as well as its interaction effect with task (Fig. 4a; Table 1). The post hoc simple slope analyses revealed that stronger FC within N_6_ was associated with lower σ_vM_ and thus higher memory precision in the orientation task (β = −0.073, *P* = 0.003), whereas no such association was found for the location task (β = −0.009, *P* = 0.701). In addition, there were also significant main and interaction effects of the between-network FC of N_2_ and N_4_ (Fig. 4b). The post hoc analyses revealed that for the orientation task, stronger FC between N_2_ and N_4_ was associated with lower σ_vM_, that is, higher memory precision (β = −0.076, *P* = 0.002), whereas for the location task, the association was not significant (β = −0.014, *P* = 0.542). It should be noted, however, that the significant interaction effect between task and the between-network FC of N_2_ and N_4_ was heavily driven by 3 influential cases. The updated model showed that with the exclusion of these cases, the interaction effect was no longer statistically significant (see Supplementary Results). Lastly, the model also showed that stronger FC between N_3_ and N_5_ was related to lower σ_vM_ and, therefore, higher memory precision (Fig. 4c), and again, this effect was significant only for the orientation task (β = −0.077, *P* = 0.002) but not the location task (β = −0.001, *P* = 0.960).

**Table 1:** The best-fitting model of memory precision.

**Fixed effects**	**Estimate**	***SEM***	***t***	**Bootstrap-based 95% CI**	***P***
**Intercept (Orientation)**	0.74	0.02	31.61	[0.69, 0.78]	1.15e-63
**Task (Location)**	−0.34	0.03	−11.28	[−0.40, −0.28]	3.22e-17
**FC**_***W***_ **of N**_**6**_	−0.07	0.02	−3.07	[−0.12, −0.03]	0.003
**FC**_***B***_ **of N**_**2**_ **and N**_**4**_	−0.08	0.02	−3.22	[−0.12, −0.03]	0.002
**FC**_***B***_ **of N**_**3**_ **and N**_**5**_	−0.08	0.02	−3.21	[−0.12, −0.03]	0.002
**Task × FC**_***W***_ **of N**_**6**_	0.06	0.03	2.07	[0.00, 0.13]	0.042
**Task × FC**_***B***_ **of N**_**2**_ **and N**_**4**_	0.06	0.03	2.01	[0.00, 0.12]	0.049
**Task × FC**_***B***_ **of N**_**3**_ **and N**_**5**_	0.08	0.03	2.43	[0.01, 0.14]	0.018

FC*_W_*, within-network FC; FC*_B_*, between-network FC.

No significant effect of within- and between-network FC was found in the model of swap errors (*Ps* > 0.105. The best-fitting model of random guesses, on the other hand, revealed significant effects of the between-network FC of N_3_ and N_5_, showing both a significant main effect and a significant interaction with task (Fig. 4d; Table 2). The post hoc analyses showed that stronger FC between N_3_ and N_5_ was associated with a higher probability of random guesses in the orientation task (β = 0.058, *P* = 0.003), whereas no such association was found for the location task (β = 0.006, *P* = 0.736).

**Table 2:** The best-fitting model of random guesses.

**Fixed effects**	**Estimate**	***SEM***	***t***	**Bootstrap-based 95% CI**	***P***
**Intercept (Orientation)**	0.36	0.02	19.45	[0.33, 0.40]	1.69e-41
**Task (Location)**	−0.34	0.03	−13.44	[−0.39, −0.29]	4.59e-21
**FC**_***B***_ **of N**_**3**_ **and N**_**5**_	0.06	0.02	3.07	[0.02, 0.10]	0.003
**Task × FC**_***B***_ **of N**_**3**_ **and N**_**5**_	−0.05	0.03	−2.02	[−0.10, 0.00]	0.048

FC*_W_*, within-network FC; FC*_B_*, between-network FC.

## Discussion

In this study, we investigated the relationships between resting-state functional networks and distinct aspects of VWM performance, including memory precision, swap errors, and random guesses. We first identified 6 intrinsic functional networks using hierarchical clustering, including a prefrontal-occipital network, a dorsal attention network, a frontoparietal control network, an angular-gyrus-cerebellar network, a cingulo-opercular network, and a frontal-opercular network. Consistent with our hypothesis, we found networks uniquely associated with memory precision; specifically, stronger FC within the frontal-opercular network and between the dorsal attention network and the angular-gyrus-cerebellar network was linked to more precise task responses. In addition, we identified intrinsic networks shared between memory precision and random guesses, where stronger FC between the frontoparietal control network and the cingulo-opercular network was associated with higher memory precision and fewer guesses. While our previous work implied that associations between brain networks and response errors were not specific to either the spatial or object VWM tasks (Li et al. 2023), the current findings indicate a contrasting pattern. Here, significant networks and patterns of network couplings were found to vary depending on the visual attributes examined—predominantly, our principal findings were specific to the orientation task.

Previous rs-fMRI studies have highlighted the role of resting-state networks in accounting for aggregate behavioral variability in VWM tasks (Sala-Llonch et al. 2012; Jolles et al. 2013; Keller et al. 2015). In this study, the use of a delayed estimation task and computational modeling, recognizing the heterogeneous nature of VWM task performance, has allowed us to identify critical networks for memory precision and random guessing separately. Unlike most prior studies that focused exclusively on a select group of canonical networks, we included not only cortical but also subcortical and cerebellar regions in our data-driven clustering analysis. This approach allows us to reproduce some well-defined networks as well as to identify networks that encompass a wider range of anatomical regions. For instance, the network N_4_ we identified comprises the bilateral angular gyri and the Crus I and lobule VII of the left cerebellum. While the angular gyrus is a region that consistently demonstrates deactivation during various tasks as a node of the default mode network (Buckner et al. 2008), the left posterior cerebellum is involved in a wide range of higher order cognitive functions, including cognitive control and spatial processing (O’Reilly et al. 2009; Stoodley and Schmahmann 2009; Strick et al. 2009). This indicates that N_4_ does not correspond to the well-established default mode network. Indeed, according to a comprehensive review (Seghier 2013), the angular gyrus is considered a critical hub for combining multisensory information with prior experiences and knowledge. The N_4_ we identified may instead contribute to the cross-modal integration of information. Our analyses further revealed that stronger coupling between N_4_ and N_2_ was associated with higher memory precision. The network N_2_ corresponds well to the dorsal attention network, which is thought to facilitate goal-directed selection over perceptual stimuli and action plans (Corbetta and Shulman 2002). This network was hypothesized to be associated with memory precision specifically. The current finding indicates that while the dorsal attention network is indeed involved, it does not act independently. Instead, to modulate memory precision, it functions in coordination with the angular-gyrus-cerebellar network. Increased FC between these networks may help strengthen the representations of specific items and features by integrating visuospatial inputs with contextual, internal task goals, which, as a result, contributes to more precise working memory responses.

In addition to N_2_ and N_4_, the network N_6_, comprising the bilateral pars orbitalis of inferior frontal gyrus and the right precentral gyrus, Rolandic operculum, and Heschl’s gyrus was also specifically associated with memory precision. Increased FC within N_6_ was associated with higher memory precision. In studies of semantic and emotional processing, the pars orbitalis of inferior frontal gyrus is consistently co-activated with the precentral gyrus (Belyk et al. 2017). Through a long-range white matter bundle, the inferior frontal gyrus is also structurally connected to the opercular cortices where the Rolandic operculum and Heschl’s gyrus lie (Briggs et al. 2019). Intrinsic FC found in this study further confirms the functional similarity of these regions, at least among a large set of ROIs that have been involved in VWM tasks. The pars orbitalis of the inferior frontal gyrus is involved in the perception of semantic meaning during communication (Belyk et al. 2017) and learning (Race et al. 2009). The precentral gyrus and Rolandic operculum are linked with speech production (Triarhou 2021; Silva et al. 2022), and Heschl’s gyrus has been associated with spontaneous inner speech (Hurlburt et al. 2016). The network N_6_ may, therefore, contribute to the articulatory processing of semantically meaningful contents. This aligns with the substantial body of evidence indicating that verbal or semantic codes are utilized in the representation of visual information such as pictures, colors, and abstract shapes (Brown et al. 2006; Mate et al. 2012; Logie et al. 2020). Individual differences in the strength of FC within this frontal-opercular network may thus reflect how well participants could recruit additional codes, potentially linguistic in nature, to encode, rehearse, and retrieve the visually presented items. Although our interpretation of the role of the frontal-opercular network seems plausible, we must acknowledge that these interpretations are speculative, as this network may also support other cognitive processes.

Besides the brain-behavior associations specific to memory precision, we found that the FC between N_3_ and N_5_ was linked to both memory precision and random guesses. Notably, stronger FC between the networks was associated with higher memory precision but, in a seemingly contradictory fashion, also with a *higher* rate of random guessing. The network N_3_ corresponds to a right-lateralized frontoparietal control network responsible for executive control (Vincent et al. 2008; Dixon et al. 2018), whereas N_5_ corresponds well to the cingulo-opercular network involved in task set implementation and switching based on rewards (Dosenbach et al. 2006; Dosenbach et al. 2007; Seeley et al. 2007; Han et al. 2018). The involvement of the cingulo-opercular network aligns with our hypothesis, which identified the network as a prime candidate for regulating both memory precision and one other type of response error. Although we predicted that the frontoparietal control network would specifically contribute to memory precision, our result indicates that it also plays a role in regulating random guesses. To guide overt behaviors, the cingulo-opercular network is believed to represent motivational incentives to regulate cognitive control processes in the frontoparietal control network (Kouneiher et al. 2009; Wood and Nee 2023). In our experiment, a feedback stimulus appeared in each trial to help participants evaluate their responses. We speculate that the enhanced couplings between the frontoparietal control network and cingulo-opercular network found in this study could facilitate the learning of associations between precise responses and specific task-performing strategies, leading to more and more such responses over trials. However, due to the growing frequency of obtaining rewards (i.e. the precise responses per se), monitoring of task performance might subside, which can result in attentional lapses or impulsive, reward-seeking behaviors that constitute random guesses. Increases in response errors detected by the cingulo-opercular network may again motivate the frontoparietal control network, which, in turn, updates the task-performing strategies to drive more precise responses. While these interpretations are tentative, it is clear from our findings that the relationship between VWM performance and intrinsic networks is complex.

Our experiment investigated the effects of visual features by incorporating tasks for recalling spatial location and object features. A recent study by Ren et al. (2019) has further distinguished between spatial and object working memory based on their patterns of resting-state FC. They found that the capacity of spatial working memory was associated with FC between the left dorsolateral prefrontal cortex and left precuneus, between the right dorsolateral prefrontal cortex and right middle frontal gyrus, and between the left superior frontal sulcus and right inferior parietal lobule. The capacity of object working memory was associated with FC between the right intraparietal sulcus and the left postcentral gyrus, left supplementary motor area, and right precentral gyrus (Ren et al. 2019). In the present study, we found task-specific brain-behavior associations, with principal findings emerging from the orientation task. This contrasts with our earlier study on white matter microstructure, which showed that associations were not specific to either location or orientation task but generalized across both (Li et al. 2023). These findings collectively suggest that in supporting VWM for object orientation, the brain relies not merely on direct interareal communication via large coherent white matter tracts but also on indirect communication through functional couplings within and between intrinsic networks.

Our results clearly demonstrate that multiple large-scale intrinsic functional networks are engaged to facilitate VWM task performance. Importantly, distinct sets of networks are employed to regulate either memory precision in isolation or both precision and random guesses. These observations support the recent theoretical model of working memory proposed by Logie et al. (2020). Under Logie et al. framework, working memory comprises multiple domain-specific temporary stores and cognitive processes that, notably, work in unison. Given this, conventional aggregate measures of working memory performance are understood to reflect the collective contributions of these stores and processes, as well as the overall efficiency with which these components operate together (Logie et al. 2020). By distinguishing between memory precision, swap errors, and random guesses, our study allows us to identify theoretical principles of the system that may have been obscured when only a single behavioral measure is used. Indeed, our findings enhance our understanding of the system’s efficiency by providing evidence of its inherent flexibility. Specifically, in response to particular task demands—namely, maintaining the fine-grained visual representations of target items or adjusting task responses to prevent guesses—the system can mobilize different combinations of cognitive processes across different functional domains.

Despite our insightful results, there are several limitations that we need to discuss. First, it is important to note that the behavioral measures estimated for the location task exhibit significantly less variability compared to those for the orientation task. This reduced variability may be because the location task is easier relative to the orientation task. A potential ceiling effect might have restricted our ability to identify associations’ specific to the location task, suggesting that we should interpret the task specificity of the results with more caution. Similarly, this ceiling effect may have limited our ability to identify brain-behavior associations for swap errors. However, it is also plausible that swap errors are modulated by neural processing within specific brain regions rather than across larger-scale networks, as indicated by feature-binding deficits observed in patients with localized brain lesions in the postcentral gyrus (Lugtmeijer et al. 2021). To better differentiate between a ceiling effect and a true null effect in future studies, the location task could be made more challenging by requiring participants to memorize gratings placed across both visual hemifields rather than only those on the cued side. Second, our selection of ROIs, combined with the data-driven hierarchical clustering, has enabled us to identify networks that were less explored in previous studies. However, some well-established networks, such as the default mode network, were not detected by our approach. The inability to identify the default mode network may have limited our ability to fully test our hypothesis since this network was hypothesized to specifically modulate random guesses. Future studies may consider including more regions or even adopting whole-brain voxel-wise approaches to address this question. Finally, our study predominantly focused on young college students. This narrow demographic focus has restricted the generalizability of our findings, as the networks related to different sources of response errors may vary significantly across age groups. Future studies might consider testing our hypotheses in children and adolescents, particularly in light of the increasing recognition of mixture distribution modeling as a valuable tool in studying cognitive development (Burnett Heyes et al. 2012; Burnett Heyes et al. 2016).

## Conclusion

In conclusion, our study demonstrates that distinct sources of error in VWM tasks are underpinned by different yet partially overlapping intrinsic functional networks. We found that higher memory precision is associated with increased connectivity within the frontal-opercular network, as well as between the dorsal attention network and the angular-gyrus-cerebellar network. Identifying neural correlates specific to memory precision implies that multiple cognitive processes are selectively recruited to maintain high-fidelity working memory representations. Additionally, we found that stronger coupling between the frontoparietal control network and the cingulo-opercular network is a common factor underlying both higher memory precision and increased random guesses, suggesting the potential role of a shared cognitive mechanism that facilitates constant adjustment of task responses.

## Supplementary Material

LI_X_Supplement_Final_bhae401

## Data Availability

The data that support the findings of this study are openly available in OSF at https://osf.io/kem24/.

## References

[ref1] Andersson JL , Hutton C, Ashburner J, Turner R, Friston K. Modeling geometric deformations in EPI time series. Neuroimage. 2001:13:903–919. 10.1006/nimg.2001.0746. 11304086

[ref2] Ashburner J , Friston KJ. Unified segmentation. Neuroimage. 2005:26:839–851. 10.1016/j.neuroimage.2005.02.018. 15955494

[ref3] Bates D , Mächler M, Bolker B, Walker S. Fitting linear mixed-effects models using lme4. J Stat Softw. 2015:67:1–48. 10.18637/jss.v067.i01.

[ref4] Bays PM . Evaluating and excluding swap errors in analogue tests of working memory. Sci Rep. 2016:6:19203. 10.1038/srep19203. 26758902 PMC4725843

[ref5] Bays PM , Catalao RF, Husain M. The precision of visual working memory is set by allocation of a shared resource. J Vis. 2009:9:7.1–7.11. 10.1167/9.10.7. 19810788 PMC3118422

[ref6] Bays PM , Gorgoraptis N, Wee N, Marshall L, Husain M. Temporal dynamics of encoding, storage, and reallocation of visual working memory. J Vis. 2011a:11:1–15. 10.1167/11.10.6. PMC3401684 21911739

[ref7] Bays PM , Wu EY, Husain M. Storage and binding of object features in visual working memory. Neuropsychologia. 2011b:49:1622–1631. 10.1016/j.neuropsychologia.2010.12.023. 21172364 PMC3119435

[ref8] Behzadi Y , Restom K, Liau J, Liu TT. A component based noise correction method (CompCor) for BOLD and perfusion based fMRI. Neuroimage. 2007:37:90–101. 10.1016/j.neuroimage.2007.04.042. 17560126 PMC2214855

[ref9] Belyk M , Brown S, Lim J, Kotz SA. Convergence of semantics and emotional expression within the IFG pars orbitalis. Neuroimage. 2017:156:240–248. 10.1016/j.neuroimage.2017.04.020. 28400265

[ref10] Benjamini Y , Hochberg Y. Controlling the false discovery rate: a practical and powerful approach to multiple testing. J R Stat Soc Ser B Methodol. 1995:57:289–300. 10.1111/j.2517-6161.1995.tb02031.x.

[ref11] Boly M , Perlbarg V, Marrelec G, Schabus M, Laureys S, Doyon J, Pelegrini-Issac M, Maquet P, Benali H. Hierarchical clustering of brain activity during human nonrapid eye movement sleep. Proc Natl Acad Sci U S A. 2012:109:5856–5861. 10.1073/pnas.1111133109. 22451917 PMC3326471

[ref12] Brainard DH . The psychophysics toolbox. Spat Vis. 1997:10:433–436. 10.1163/156856897X00357. 9176952

[ref13] Briggs RG , Chakraborty AR, Anderson CD, Abraham CJ, Palejwala AH, Conner AK, Pelargos PE, O'Donoghue DL, Glenn CA, Sughrue ME. Anatomy and white matter connections of the inferior frontal gyrus. Clin Anat. 2019:32:546–556. 10.1002/ca.23349. 30719769

[ref14] Brown LA , Forbes D, McConnell J. Limiting the use of verbal coding in the visual patterns test. Q J Exp Psychol (Hove). 2006:59:1169–1176. 10.1080/17470210600665954. 16769617

[ref15] Buckner RL , Andrews-Hanna JR, Schacter DL. The Brain's default network: anatomy, function, and relevance to disease. Ann N Y Acad Sci. 2008:1124:1–38. 10.1196/annals.1440.011. 18400922

[ref16] Burnett Heyes S , Zokaei N, van der Staaij I, Bays PM, Husain M. Development of visual working memory precision in childhood. Dev Sci. 2012:15:528–539. 10.1111/j.1467-7687.2012.01148.x. 22709402 PMC3401951

[ref17] Burnett Heyes S , Zokaei N, Husain M. Longitudinal development of visual working memory precision in childhood and early adolescence. Cogn Dev. 2016:39:36–44. 10.1016/j.cogdev.2016.03.004. 27546982 PMC4981317

[ref18] Chai XJ , Castañón AN, Öngür D, Whitfield-Gabrieli S. Anticorrelations in resting state networks without global signal regression. Neuroimage. 2012:59:1420–1428. 10.1016/j.neuroimage.2011.08.048. 21889994 PMC3230748

[ref19] Christoff K , Irving ZC, Fox KC, Spreng RN, Andrews-Hanna JR. Mind-wandering as spontaneous thought: a dynamic framework. Nat Rev Neurosci. 2016:17:718–731. 10.1038/nrn.2016.113. 27654862

[ref20] Corbetta M , Shulman GL. Control of goal-directed and stimulus-driven attention in the brain. Nat Rev Neurosci. 2002:3:201–215. 10.1038/nrn755. 11994752

[ref21] Cordes D , Haughton V, Carew JD, Arfanakis K, Maravilla K. Hierarchical clustering to measure connectivity in fMRI resting-state data. Magn Reson Imaging. 2002:20:305–317. 10.1016/s0730-725x(02)00503-9. 12165349

[ref22] Damoiseaux JS , Rombouts SA, Barkhof F, Scheltens P, Stam CJ, Smith SM, Beckmann CF. Consistent resting-state networks across healthy subjects. Proc Natl Acad Sci U S A. 2006:103:13848–13853. doi:10.1073/pnas.0601417103. 16945915 PMC1564249

[ref23] Dixon ML , De La Vega A, Mills C, Andrews-Hanna J, Spreng RN, Cole MW, Christoff K. Heterogeneity within the frontoparietal control network and its relationship to the default and dorsal attention networks. Proc Natl Acad Sci U S A. 2018:115:E1598–E1607. doi:10.1073/pnas.1715766115. 29382744 PMC5816169

[ref24] Dosenbach NU , Visscher KM, Palmer ED, Miezin FM, Wenger KK, Kang HC, Burgund ED, Grimes AL, Schlaggar BL, Petersen SE. A core system for the implementation of task sets. Neuron. 2006:50:799–812. 10.1016/j.neuron.2006.04.031. 16731517 PMC3621133

[ref25] Dosenbach NU , Fair DA, Miezin FM, Cohen AL, Wenger KK, Dosenbach RA, Fox MD, Snyder AZ, Vincent JL, Raichle ME, et al. Distinct brain networks for adaptive and stable task control in humans. Proc Natl Acad Sci U S A. 2007:104:11073–11078. 10.1073/pnas.0704320104. 17576922 PMC1904171

[ref26] Fisher NI . Statistical analysis of circular data. Cambridge: Cambridge University Press; 1995.

[ref27] Fox MD , Snyder AZ, Vincent JL, Corbetta M, Van Essen DC, Raichle ME. The human brain is intrinsically organized into dynamic, anticorrelated functional networks. Proc Natl Acad Sci U S A. 2005:102:9673–9678. 10.1073/pnas.0504136102. 15976020 PMC1157105

[ref28] Friston KJ , Williams S, Howard R, Frackowiak RS, Turner R. Movement-related effects in fMRI time-series. Magn Reson Med. 1996:35:346–355. 10.1002/mrm.1910350312. 8699946

[ref29] Hallquist MN , Hwang K, Luna B. The nuisance of nuisance regression: spectral misspecification in a common approach to resting-state fMRI preprocessing reintroduces noise and obscures functional connectivity. Neuroimage. 2013:82:208–225. 10.1016/j.neuroimage.2013.05.116. 23747457 PMC3759585

[ref30] Han SW , Eaton HP, Marois R. Functional fractionation of the cingulo-opercular network: alerting insula and updating cingulate. Cereb Cortex. 2018:29:2624–2638. 10.1093/cercor/bhy130. PMC7963117 29850839

[ref31] Henson R , Büechel C, Josephs O, Friston K. The slice-timing problem in event-related fMRI. Neuroiage. 1999:9:125.

[ref32] Hurlburt RT , Alderson-Day B, Kühn S, Fernyhough C. Exploring the ecological validity of thinking on demand: neural correlates of elicited vs. spontaneously occurring inner speech. PLoS One. 2016:11:e0147932. 10.1371/journal.pone.0147932. 26845028 PMC4741522

[ref33] Jafri MJ , Pearlson GD, Stevens M, Calhoun VD. A method for functional network connectivity among spatially independent resting-state components in schizophrenia. Neuroimage. 2008:39:1666–1681. 10.1016/j.neuroimage.2007.11.001. 18082428 PMC3164840

[ref34] Jolles DD , van Buchem MA, Crone EA, Rombouts SA. Functional brain connectivity at rest changes after working memory training. Hum Brain Mapp. 2013:34:396–406. 10.1002/hbm.21444. 22076823 PMC6870317

[ref35] Kassambara A . rstatix: pipe-friendly framework for basic statistical tests. R package version 0.6. 0. 2020.

[ref36] Keller JB , Hedden T, Thompson TW, Anteraper SA, Gabrieli JD, Whitfield-Gabrieli S. Resting-state anticorrelations between medial and lateral prefrontal cortex: association with working memory, aging, and individual differences. Cortex. 2015:64:271–280. 10.1016/j.cortex.2014.12.001. 25562175 PMC4346444

[ref37] Kouneiher F , Charron S, Koechlin E. Motivation and cognitive control in the human prefrontal cortex. Nat Neurosci. 2009:12:939–945. 10.1038/nn.2321. 19503087

[ref38] Kuznetsova A , Brockhoff PB, Christensen RHB. lmerTest package: tests in linear mixed effects models. J Stat Softw. 2017:82:1–26. 10.18637/jss.v082.i13.

[ref39] Li X , O'Sullivan MJ, Mattingley JB. Delay activity during visual working memory: a meta-analysis of 30 fMRI experiments. Neuroimage. 2022:255:119204. 10.1016/j.neuroimage.2022.119204. 35427771

[ref40] Li X , Rangelov D, Mattingley JB, Oestreich L, Levy-Bencheton D, O'Sullivan MJ. White matter microstructure is associated with the precision of visual working memory. Neuroimage. 2023:272:120069. 10.1016/j.neuroimage.2023.120069. 37003445

[ref41] Logie RH . Visuo-spatial processing in working memory. J Q Exp Psychol A. 1986:38:229–247. 10.1080/14640748608401596. 3737975

[ref42] Logie RH . Visuo-spatial working memory. Hillsdale (NJ): Lawrence Erlbaum Associates, Inc.; 1995.

[ref43] Logie RH , Belletier C, Doherty JM. Integrating theories of working memory. In: Logie R, Camos V, Cowan N, editors. Working memory: the state of the science. Oxford: Oxford University Press; 2020, p. 389–430. 10.1093/oso/9780198842286.003.0014

[ref44] Long JA . Interactions: comprehensive, user-friendly toolkit for probing interactions. In R package version 1.1.6. 2022. https://cran.r-project.org/package=interactions.

[ref45] Lugtmeijer S , Schneegans S, Lammers NA, Geerligs L, de Leeuw FE, de Haan EHF, Bays PM, Kessels RPC. Consequence of stroke for feature recall and binding in visual working memory. Neurobiol Learn Mem. 2021:179:107387. 10.1016/j.nlm.2021.107387. 33460791

[ref79] Ma WJ , Husain M, Bays PM. Changing concepts of working memory. *Nat Neurosci*. 2014:17(3):47–356. 10.1038/nn.3655. PMC4159388 24569831

[ref46] Marques JP , Kober T, Krueger G, van der Zwaag W, Van de Moortele PF, Gruetter R. MP2RAGE, a self bias-field corrected sequence for improved segmentation and T1-mapping at high field. Neuroimage. 2010:49:1271–1281. 10.1016/j.neuroimage.2009.10.002. 19819338

[ref47] Massey FJ . The Kolmogorov-Smirnov test for goodness of fit. J Am Stat Assoc. 1951:46:68–78. 10.1080/01621459.1951.10500769.

[ref48] Mate J , Allen RJ, Baqués J. What you say matters: exploring visual–verbal interactions in visual working memory. Q J Exp Psychol (Hove). 2012:65:395–400. 10.1080/17470218.2011.644798. 22248026

[ref49] Nieto-Castanon A . Handbook of functional connectivity magnetic resonance imaging methods in CONN. Boston (MA): Hilbert Press; 2020. 10.56441/hilbertpress.2207.6598.

[ref50] Nieuwenhuis R , Te Grotenhuis H, Pelzer B. Influence. ME: tools for detecting influential data in mixed effects models. R J. 2012:4:38–47. 10.32614/RJ-2012-011.

[ref51] O'Reilly JX , Beckmann CF, Tomassini V, Ramnani N, Johansen-Berg H. Distinct and overlapping functional zones in the cerebellum defined by resting state functional connectivity. Cereb Cortex. 2009:20:953–965. 10.1093/cercor/bhp157. 19684249 PMC2837094

[ref52] Pelli DG . The VideoToolbox software for visual psychophysics: transforming numbers into movies. Spat Vis. 1997:10:437–442. 10.1163/156856897X00366. 9176953

[ref53] Power JD , Mitra A, Laumann TO, Snyder AZ, Schlaggar BL, Petersen SE. Methods to detect, characterize, and remove motion artifact in resting state fMRI. Neuroimage. 2014:84:320–341. 10.1016/j.neuroimage.2013.08.048. 23994314 PMC3849338

[ref54] Pratte MS , Park YE, Rademaker RL, Tong F. Accounting for stimulus-specific variation in precision reveals a discrete capacity limit in visual working memory. J Exp Psychol Hum Percept Perform. 2017:43:6–17. 10.1037/xhp0000302. 28004957 PMC5189913

[ref55] Race EA , Shanker S, Wagner AD. Neural priming in human frontal cortex: multiple forms of learning reduce demands on the prefrontal executive system. J Cogn Neurosci. 2009:21:1766–1781. 10.1162/jocn.2009.21132. 18823245 PMC2788302

[ref56] Ren Z , Zhang Y, He H, Feng Q, Bi T, Qiu J. The different brain mechanisms of object and spatial working memory: voxel-based morphometry and resting-state functional connectivity. Front Hum Neurosci. 2019:13:248. 10.3389/fnhum.2019.00248. 31379543 PMC6659551

[ref57] Sala-Llonch R , Pena-Gomez C, Arenaza-Urquijo EM, Vidal-Pineiro D, Bargallo N, Junque C, Bartres-Faz D. Brain connectivity during resting state and subsequent working memory task predicts behavioural performance. Cortex. 2012:48:1187–1196. 10.1016/j.cortex.2011.07.006. 21872853

[ref58] Salvador R , Suckling J, Coleman MR, Pickard JD, Menon D, Bullmore E. Neurophysiological architecture of functional magnetic resonance images of human brain. Cereb Cortex. 2005:15:1332–1342. 10.1093/cercor/bhi016. 15635061

[ref59] Schneegans S , Bays PM. No fixed item limit in visuospatial working memory. Cortex. 2016:83:181–193. 10.1016/j.cortex.2016.07.021. 27565636 PMC5043407

[ref60] Schneegans S , Bays PM. Neural architecture for feature binding in visual working memory. J Neurosci. 2017:37:3913–3925. 10.1523/JNEUROSCI.3493-16.2017. 28270569 PMC5394900

[ref61] Seeley WW , Menon V, Schatzberg AF, Keller J, Glover GH, Kenna H, Reiss AL, Greicius MD. Dissociable intrinsic connectivity networks for salience processing and executive control. J Neurosci. 2007:27:2349–2356. 10.1523/JNEUROSCI.5587-06.2007. 17329432 PMC2680293

[ref62] Seghier ML . The angular gyrus: multiple functions and multiple subdivisions. Neuroscientist. 2013:19:43–61. 10.1177/1073858412440596. 22547530 PMC4107834

[ref63] Silva AB , Liu JR, Zhao L, Levy DF, Scott TL, Chang EF. A neurosurgical functional dissection of the middle precentral gyrus during speech production. J Neurosci. 2022:42:8416–8426. 10.1523/JNEUROSCI.1614-22.2022. 36351829 PMC9665919

[ref64] Sladky R , Friston KJ, Tröstl J, Cunnington R, Moser E, Windischberger C. Slice-timing effects and their correction in functional MRI. Neuroimage. 2011:58:588–594. 10.1016/j.neuroimage.2011.06.078. 21757015 PMC3167249

[ref65] Sorensen TA . A method of establishing groups of equal amplitude in plant sociology based on similarity of species content and its application to analyses of the vegetation on Danish commons. Biol Skar. 1948:5:1–34.

[ref66] Stoodley CJ , Schmahmann JD. Functional topography in the human cerebellum: a meta-analysis of neuroimaging studies. Neuroimage. 2009:44:489–501. 10.1016/j.neuroimage.2008.08.039. 18835452

[ref67] Strick PL , Dum RP, Fiez JA. Cerebellum and nonmotor function. Annu Rev Neurosci. 2009:32:413–434. 10.1146/annurev.neuro.31.060407.125606. 19555291

[ref68] Triarhou LC . Cytoarchitectonics of the Rolandic operculum: morphofunctional ponderings. Brain Struct Funct. 2021:226:941–950. 10.1007/s00429-021-02258-z. 33743075

[ref70] Vincent JL , Kahn I, Snyder AZ, Raichle ME, Buckner RL. Evidence for a frontoparietal control system revealed by intrinsic functional connectivity. J Neurophysiol. 2008:100:3328–3342. 10.1152/jn.90355.2008. 18799601 PMC2604839

[ref71] Vossel S , Geng JJ, Fink GR. Dorsal and ventral attention systems: distinct neural circuits but collaborative roles. Neuroscientist. 2014:20:150–159. 10.1177/1073858413494269. 23835449 PMC4107817

[ref72] Wang Y , Li TQ. Analysis of whole-brain resting-state FMRI data using hierarchical clustering approach. PLoS One. 2013:8:e76315. 10.1371/journal.pone.0076315. 24204612 PMC3799854

[ref73] White CN , Servant M, Logan GD. Testing the validity of conflict drift-diffusion models for use in estimating cognitive processes: a parameter-recovery study. Psychon Bull Rev. 2018:25:286–301. 10.3758/s13423-017-1271-2. 28357629 PMC5788738

[ref74] Wilson RC , Collins AG. Ten simple rules for the computational modeling of behavioral data. Elife. 2019:8:e49547. 10.7554/eLife.49547. PMC6879303 31769410

[ref75] Wood JL , Nee DE. Cingulo-Opercular subnetworks motivate Frontoparietal subnetworks during distinct cognitive control demands. J Neurosci. 2023:43:1225–1237. 10.1523/JNEUROSCI.1314-22.2022. 36609452 PMC9962782

[ref76] Yeo BT , Krienen FM, Sepulcre J, Sabuncu MR, Lashkari D, Hollinshead M, Roffman JL, Smoller JW, Zollei L, Polimeni JR, et al. The organization of the human cerebral cortex estimated by intrinsic functional connectivity. J Neurophysiol. 2011:106:1125–1165. 10.1152/jn.00338.2011. 21653723 PMC3174820

[ref77] Zhang W , Luck SJ. Discrete fixed-resolution representations in visual working memory. Nature. 2008:453:233–235. 10.1038/nature06860. 18385672 PMC2588137

[ref78] Zhou C , Zemanova L, Zamora G, Hilgetag CC, Kurths J. Hierarchical organization unveiled by functional connectivity in complex brain networks. Phys Rev Lett. 2006:97:238103. 10.1103/PhysRevLett.97.238103. 17280251

