# Supplementary material for: Intrinsic functional networks for distinct sources of error in visual working memory
Source: Cereb Cortex. 2024 Oct 9;34(10):bhae401. doi: 10.1093/cercor/bhae401 (PMC11464681; doi:10.1093/cercor/bhae401)
Supplement: LI_X_Supplement_Final_bhae401 [file li_x_supplement_final_bhae401.docx]

**Intrinsic connectivity networks for distinct sources of error in visual working memory**

**Supplementary Materials**

**Supplementary Methods.** Recoverability of Model Parameters.

**Supplementary Results.** Analysis of Influential Cases in Mixed Model.

**Table S1.** Hierarchical Clustering of Regions of Interest.

**Table S2.** Multivariate Parametric Tests and Group-level Correlations.

**Table S3.** Model Comparison and Selection: Memory Precision.

**Table S4.** Model Comparison and Selection: Swap Errors.

**Table S5.** Model Comparison and Selection: Random Guesses.

**Supplementary Methods.** Recoverability of Model Parameters

We have performed analyses to assess the recoverability of the model parameters. We conducted a parameter recovery simulation, where we generated surrogate data from known parameter values, then fitted the model to this surrogate data, and compared the estimated parameters to their true values. Ideally, the estimated and true parameter values would exhibit a strong correlation.

Simulations for the mixture component model were conducted separately for each task. For each simulation, 250 surrogate datasets were generated, each containing 240 simulated trials, matching the number of trials in our empirical experiment. The parameters for each dataset were randomly generated from a uniform distribution covering a range of plausible values for each model parameter as recommended in a recent guideline article (Wilson and Collins 2019). Specifically, for the orientation task, the parameter ranges were κ = 1**–**12, P*_G_* = 0**–**0.6, and P*_NT_* = 0**–**0.3. Similarly, for the location task, the parameter ranges were κ = 1**–**12, P*_G_* = 0**–**0.6, and P*_NT_* = 0**–**0.3. These values were chosen to match the ranges of the parameter values obtained from fitting real participants’ data. To evaluate parameter recovery, we calculated the Pearson’s correlation between the true and estimated parameter values. According to White et al. (2018), parameter recovery is considered as poor for correlation coefficient (*r*) below 0.5, fair for *r* between 0.5 and 0.75, good for *r* between 0.75 and 0.9, and excellent for *r* exceeding 0.9.

For the orientation task, the von Mises concentration parameter κ (*r* = 0.89), the probability of swap errors P*_NT_* (*r* = 0.82), and the probability of random guesses P*_G_* (*r* = 0.86) all met the predefined criteria for good recovery (White et al. 2018). For the location task, the recovery of P*_G_* (*r* = 0.89) was good, while κ (*r* = 0.93) and P*_NT_* (*r* = 0.97) were recovered to excellent levels.

In response to reviewer suggestion, we calculated the correlations between the orientation and location tasks for the model’s fitted parameters. The correlation coefficients for the swap errors P*_NT_* and random guesses P*_G_* parameters were significant: *r_PNT_* = 0.26, *p* = 0.028 and *r_PG_* = 0.24, *p* = 0.042. The correlation for the von Mises concentration parameter κ was relatively low, *r*_κ_ = 0.16, *p* = 0.170. These results suggest that while the mixture distribution model shows the ability to recover parameters across the orientation and location tasks, it also captures task-specific differences effectively.

**Supplementary Results.** Analysis of Influential Cases in Mixed Model.

As shown in *Figure 4b*, the linear effect of FC*_B_* of N_2_ and N_4_ on memory precision could be driven by specific cases. To address this concern, we used the ‘influence.ME’ R package to detect potentially influential cases in our mixed-effects model for memory precision (Nieuwenhuis et al. 2022). The analyses involved iteratively excluding individual cases from the model to evaluate their influence on the parameter estimates. We applied standardised measures of influence, including DFBETAS and Cook’s Distance, to assess the impact on the model. Cases were considered overtly influential if DFBETAS exceeded $2/\sqrt{n}$ and Cook’s Distance was greater than $4/n$, where *n* is the number of participants in this study.

We first detected influential cases affecting the fixed effect of FC*_B_* of N_2_ and N_4_ on memory precision. As illustrated in *Panel a* of the figure below, while several cases exceeded the thresholds for DFBETAS and Cook’s distance, participants 11 and 37 were particularly notable. To assess their impact further, we excluded these individuals from the model. The re-estimated mixed-effects model, summarized below in *Table R2*, shows that the fixed effect of FC*_B_* of N_2_ and N_4_ remains statistically significant, even after the removal of these participants.


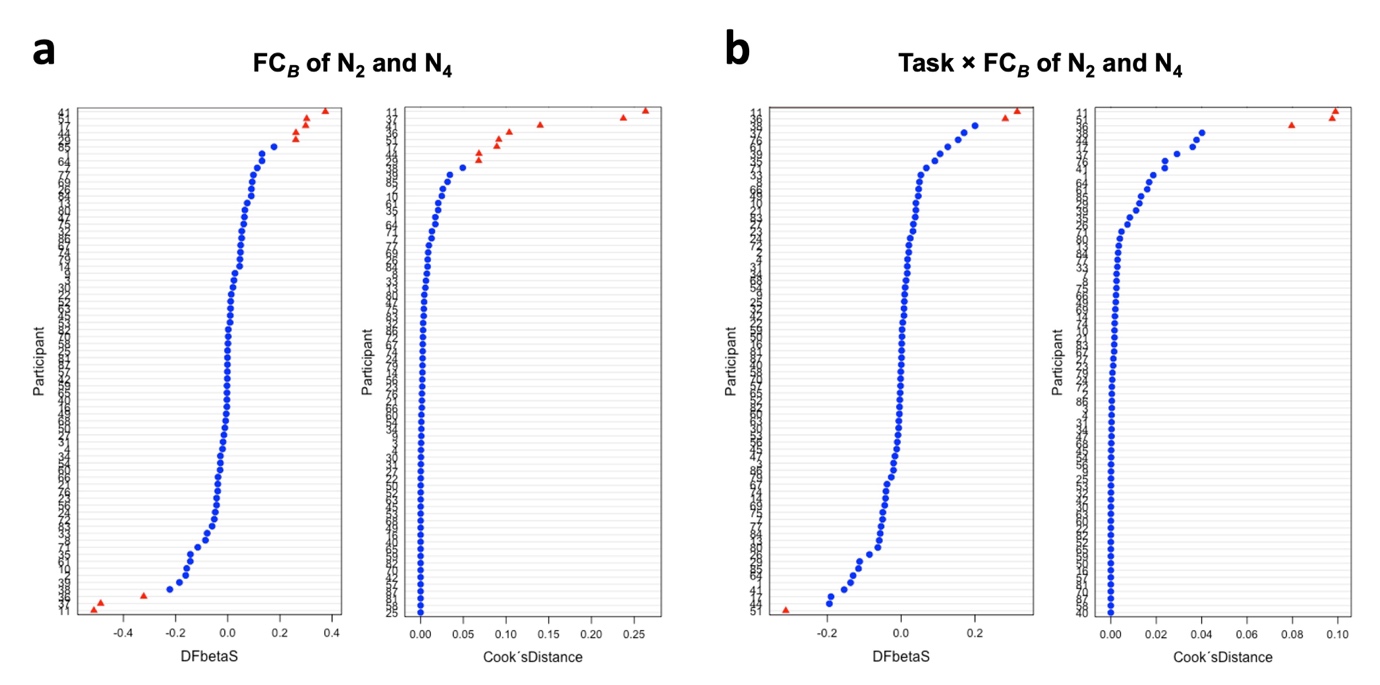


**Table R1.** The Best-fitting Model of Memory Precision: Original.

| Fixed Effects | Estimate | *SEM* | *t* | Bootstrap-based 95% CI | *p* |  |
| --- | --- | --- | --- | --- | --- | --- |
| Intercept (Orientation) | 0.74 | 0.02 | 31.61 | [0.69, 0.78] | 1.15e-63 | *** |
| Task (Location) | -0.34 | 0.03 | -11.28 | [-0.40, -0.28] | 3.22e-17 | *** |
| FC*_W_* of N_6_ | -0.07 | 0.02 | -3.07 | [-0.12, -0.03] | 0.003 | ** |
| FC*_B_* of N_2_ and N_4_ | -0.08 | 0.02 | -3.22 | [-0.12, -0.03] | 0.002 | ** |
| FC*_B_* of N_3_ and N_5_ | -0.08 | 0.02 | -3.21 | [-0.12, -0.03] | 0.002 | ** |
| Task × FC*_W_* of N_6_ | 0.06 | 0.03 | 2.07 | [0.00, 0.13] | 0.042 | * |
| Task × FC*_B_* of N_2_ and N_4_ | 0.06 | 0.03 | 2.01 | [0.00, 0.12] | 0.049 | * |
| Task × FC*_B_* of N_3_ and N_5_ | 0.08 | 0.03 | 2.43 | [0.01, 0.14] | 0.018 | * |

*Note:* FC*_W_*, within-network FC; FC*_B_*, between-network FC.

**Table R2.** The Best-fitting Model of Memory Precision: Influential Cases Excluded for the Fixed Effect of FC*_B_* of N_2_ and N_4_.

| Fixed Effects | Estimate | *SEM* | *t* | Bootstrap-based 95% CI | *p* |  |
| --- | --- | --- | --- | --- | --- | --- |
| Intercept (Orientation) | 0.72 | 0.02 | 32.07 | [0.68, 0.77] | < 0.001 | *** |
| Task (Location) | -0.33 | 0.03 | -11.02 | [-0.39, -0.27] | < 0.001 | *** |
| FC*_W_* of N_6_ | -0.07 | 0.02 | -3.03 | [-0.11, -0.02] | 0.003 | ** |
| FC*_B_* of N_2_ and N_4_ | -0.05 | 0.02 | -2.14 | [-0.10, 0.00] | 0.034 | * |
| FC*_B_* of N_3_ and N_5_ | -0.05 | 0.02 | -2.35 | [-0.10, -0.01] | 0.020 | * |
| Task × FC*_W_* of N_6_ | 0.06 | 0.03 | 1.98 | [0.00, 0.12] | 0.052 | . |
| Task × FC*_B_* of N_2_ and N_4_ | 0.05 | 0.03 | 1.43 | [-0.02, 0.11] | 0.156 |  |
| Task × FC*_B_* of N_3_ and N_5_ | 0.06 | 0.03 | 1.87 | [0.00, 0.12] | 0.066 | . |

*Note:* FC*_W_*, within-network FC; FC*_B_*, between-network FC.

We then identified potential influential cases for the interaction effect between task and FC*_B_* of N_2_ and N_4_. As illustrated in *Panel b* of the figure, participants 11, 36, and 51 appear to have a strong influence on the estimate of the interaction effect. We subsequently removed these cases and re-estimated the mixed-effects model. The updated analysis shows that (*Table R3*), with their exclusion, the interaction effect is no longer statistically significant, suggesting that the effect of FC*_B_* of N_2_ and N_4_ is not specific to either the orientation or location task.

**Table R3.** The Best-fitting Model of Memory Precision: Influential Cases Excluded for the Fixed Effect of Task × FC*_B_* of N_2_ and N_4_.

| Fixed Effects | Estimate | *SEM* | *t* | Bootstrap-based 95% CI | *p* |  |
| --- | --- | --- | --- | --- | --- | --- |
| Intercept (Orientation) | 0.72 | 0.02 | 32.02 | [0.68, 0.77] | < 0.001 | *** |
| Task (Location) | -0.33 | 0.03 | -11.30 | [-0.39, -0.27] | < 0.001 | *** |
| FC*_W_* of N_6_ | -0.05 | 0.02 | -2.23 | [-0.10, -0.01] | 0.028 | * |
| FC*_B_* of N_2_ and N_4_ | -0.06 | 0.02 | -2.67 | [-0.11, -0.02] | 0.009 | ** |
| FC*_B_* of N_3_ and N_5_ | -0.06 | 0.02 | -2.44 | [-0.10, -0.01] | 0.016 | * |
| Task × FC*_W_* of N_6_ | 0.04 | 0.03 | 1.36 | [-0.02, 0.10] | 0.179 |  |
| Task × FC*_B_* of N_2_ and N_4_ | 0.05 | 0.03 | 1.71 | [-0.01, 0.11] | 0.092 | . |
| Task × FC*_B_* of N_3_ and N_5_ | 0.06 | 0.03 | 1.97 | [0.00, 0.12] | 0.053 | . |

*Note:* FC*_W_*, within-network FC; FC*_B_*, between-network FC.

It should be noted that, despite changes in the results after controlling for influential cases, the fixed effect of FC*_B_* of N_2_ and N_4_ remains significant. Therefore, our overall interpretation and conclusions regarding the impact of N_2_ and N_4_ on memory precision do not change. We should, however, cautiously interpret the task specificity of the observed association.

**Table S1.** Hierarchical Clustering of Regions of Interest.

| Network | AAL Labels | MNI Coordinates |
| --- | --- | --- |
| 1 | L Middle frontal gyrus | -30, 2, 54 |
|  | L Superior frontal gyrus | -20, 8, 56 |
|  | L Middle occipital gyrus | -32, -78, 28 |
| 2 | L Inferior frontal gyrus pars opercularis | -46, 4, 26 |
|  | L Inferior parietal gyrus | -42, -36, 42 |
|  | L Precentral gyrus | -50, 2, 38 |
|  | L Precuneus | -14, -60, 58 |
|  | L Superior parietal gyrus | -28, -50, 58 |
|  | R Inferior parietal gyrus | 32, -46, 50 |
|  | R Inferior temporal gyrus | 54, -56, -4 |
|  | R Postcentral gyrus | 54, -24, 44 |
|  | R Superior frontal gyrus | 24, 2, 54 |
|  | R Superior parietal gyrus | 26, -60, 54 |
|  | L Superior occipital gyrus | -24, -74, 34 |
| 3 | R Inferior frontal gyrus pars opercularis | 52, 14, 20 |
|  | R Inferior frontal gyrus pars triangularis | 46, 28, 26 |
|  | R Middle frontal gyrus | 46, 36, 20 |
|  | R Supramarginal gyrus | 44, -42, 42 |
| 4 | L Angular gyrus | -42, -58, 44 |
|  | R Angular gyrus | 40, -66, 40 |
|  | L Cerebellar Crus I | -32, -66, -32 |
|  | L Cerebellar VII | -36, -56, -44 |
| 5 | R Insula | 32, 18, -10 |
|  | R Supplementary motor area | 4, 22, 58 |
|  | L Anterior cingulate cortex | 0, 24, 26 |
|  | L Insula | -36, 14, -6 |
|  | L Dorsomedial superior frontal gyrus | -6, 20, 40 |
|  | L Spplementary motor area | -6, 12, 44 |
|  | R Middle cingulate cortex | 8, 26, 32 |
|  | L Cerebellar VI | -34, -56, -28 |
|  | L Striatum | -20, 6, 4 |
|  | R Putamen | 32, 10, 2 |
| 6 | L Inferior frontal gyrus pars orbitalis | -40, 28, -6 |
|  | R Inferior frontal gyrus pars orbitalis | 42, 26, -10 |
|  | R Precentral gyrus | 46, -6, 44 |
|  | R Heschl’s gyrus | 40, -24, 16 |
|  | R Rolandic operculum | 52, -12, 12 |

*Notes:* L, left; R, right.

**Table S2.** Multivariate Parametric Tests and Group-level Correlations.

| Network Pair | |  | Multivariate Parametric Statistics | |  | Group-level FC | |
| --- | --- | --- | --- | --- | --- | --- | --- |
|  | |  | *F* (3, 69) | FDR-corrected Cluster-level *p* |  | FC*_W_* (*M*/*SEM*) | FC*_B_* (*M*/*SEM*) |
| 1 | 1 |  | 47.30 | < 0.001 |  | 0.23/0.02 |  |
|  | 2 |  | 86.25 | < 0.001 |  |  | 0.14/0.01 |
|  | 3 |  | 21.10 | < 0.001 |  |  | 0.10/0.02 |
|  | 4 |  | 25.27 | < 0.001 |  |  | 0.08/0.01 |
|  | 5 |  | 72.97 | < 0.001 |  |  | 0.05/0.01 |
|  | 6 |  | 13.04 | < 0.001 |  |  | -0.01/0.01 |
| 2 | 2 |  | 200.80 | < 0.001 |  | 0.24/0.01 |  |
|  | 3 |  | 102.76 | < 0.001 |  |  | 0.14/0.01 |
|  | 4 |  | 20.71 | < 0.001 |  |  | 0.01/0.01 |
|  | 5 |  | 71.81 | < 0.001 |  |  | 0.04/0.01 |
|  | 6 |  | 19.09 | < 0.001 |  |  | 0.04/0.01 |
| 3 | 3 |  | 125.46 | < 0.001 |  | 0.43/0.02 |  |
|  | 4 |  | 86.92 | < 0.001 |  |  | 0.19/0.02 |
|  | 5 |  | 48.00 | < 0.001 |  |  | 0.10/0.01 |
|  | 6 |  | 22.65 | < 0.001 |  |  | 0.02/0.01 |
| 4 | 4 |  | 108.34 | < 0.001 |  | 0.22/0.01 |  |
|  | 5 |  | 109.79 | < 0.001 |  |  | 0.02/0.01 |
|  | 6 |  | 24.17 | < 0.001 |  |  | -0.04/0.01 |
| 5 | 5 |  | 241.96 | < 0.001 |  | 0.21/0.01 |  |
|  | 6 |  | 69.05 | < 0.001 |  |  | 0.08/0.01 |
| 6 | 6 |  | 92.75 | < 0.001 |  | 0.14/0.01 |  |

*Notes:* FC*_W_*, within-network FC; FC*_B_*, between-network FC.

**Table S3.** Model Comparison and Selection: Memory Precision.

| Iteration | Model | Nested Model | Effect Added | REML Fit | |  | ML Fit | | | | |  | LRT Against Nested | | |
| --- | --- | --- | --- | --- | --- | --- | --- | --- | --- | --- | --- | --- | --- | --- | --- |
|  |  |  |  | LL | Order |  | *df* | AIC | BIC | LL | Deviance |  | χ^2^ | *df* | *p* |
| 1 | *Base* | - | - | 13.15 | - |  | 4 | -29.36 | -17.48 | 18.68 | -37.36 |  | - | - | - |
|  | 1 | *Base* | FC*_W_* of N_6_ + Task × FC*_W_* of N_6_ | 13.60 | 1 |  | 6 | -37.59 | -19.78 | 24.80 | -49.59 |  | 12.24 | 2 | 0.002 |
|  |  |  |  |  |  |  |  |  |  |  |  |  |  |  |  |
| 2 | 2 | *Base +* FC*_W_* of N_6_ + Task × FC*_W_* of N_6_ | - | 13.60 | 1 |  | 6 | -37.59 | -19.78 | 24.80 | -49.59 |  | 0.00 | 0 | - |
|  | 3 |  | FC*_B_* of N_2_ & N_4_ | 12.96 | 2 |  | 7 | -40.64 | -19.85 | 27.32 | -54.64 |  | 5.05 | 1 | 0.025 |
|  |  |  |  |  |  |  |  |  |  |  |  |  |  |  |  |
| 3 | 4 | *Base +* FC*_W_* of N_6_ + Task × FC*_W_* of N_6_ + FC*_B_* of N_2_ & N_4_ | - | 12.96 | 1 |  | 7 | -40.64 | -19.85 | 27.32 | -54.64 |  | 0.00 | 0 | - |
|  | 5 |  | FC*_B_* of N_3_ & N_5_ | 12.13 | 2 |  | 8 | -43.36 | -19.60 | 29.68 | -59.36 |  | 4.72 | 1 | 0.030 |
|  |  |  |  |  |  |  |  |  |  |  |  |  |  |  |  |
| 4 | 6 | *Base +* FC*_W_* of N_6_ + Task × FC*_W_* of N_6_ + FC*_B_* of N_2_ & N_4_ + FC*_B_* of N_3_ & N_5_ | - | 12.13 | 1 |  | 8 | -43.36 | -19.60 | 29.68 | -59.36 |  | 0.00 | 0 | - |
|  | 7 |  | Task × FC*_B_* of N_3_ & N_5_ | 11.90 | 2 |  | 9 | -46.13 | -19.40 | 32.06 | -64.13 |  | 4.77 | 1 | 0.029 |
|  |  |  |  |  |  |  |  |  |  |  |  |  |  |  |  |
| 5 | 8 | *Base +* FC*_W_* of N_6_ + Task × FC*_W_* of N_6_ + FC*_B_* of N_2_ & N_4_ + FC*_B_* of N_3_ & N_5_ + Task × FC*_B_* of N_3_ & N_5_ | - | 11.90 | 1 |  | 9 | -46.13 | -19.40 | 32.06 | -64.13 |  | 0.00 | 0 | - |
|  | 9 |  | Task × FC*_B_* of N_2_ & N_4_ | 11.32 | 2 |  | 10 | -48.28 | -18.58 | 34.14 | -68.28 |  | 4.15 | 1 | 0.042 |
|  |  |  |  |  |  |  |  |  |  |  |  |  |  |  |  |
| 6 | 10 | *Base +* FC*_W_* of N_6_ + Task × FC*_W_* of N_6_ + FC*_B_* of N_2_ & N_4_ + Task × FC*_B_* of N_2_ & N_4_ + FC*_B_* of N_3_ & N_5_ + Task × FC*_B_* of N_3_ & N_5_ | - | 11.32 | 1 |  | 10 | -48.28 | -18.58 | 34.14 | -68.28 |  | 0.00 | 0 | - |
|  | 11 |  | FC*_W_* of N_3_ | 9.63 | 2 |  | 11 | -49.22 | -16.55 | 35.61 | -71.22 |  | 2.94 | 1 | 0.086 |
|  | 12 |  | FC*_W_* of N_5_ | 8.95 | 3 |  | 11 | -47.70 | -15.04 | 34.85 | -69.70 |  | 1.43 | 1 | 0.232 |
|  | 13 |  | FC*_B_* of N_4_ & N_5_ | 8.93 | 4 |  | 11 | -47.66 | -14.99 | 34.83 | -69.66 |  | 1.38 | 1 | 0.240 |
|  | 14 |  | FC*_B_* of N_2_ & N_5_ | 8.72 | 5 |  | 11 | -47.30 | -14.63 | 34.65 | -69.30 |  | 1.02 | 1 | 0.313 |
|  | 15 |  | FC*_B_* of N_1_ & N_6_ | 8.67 | 6 |  | 11 | -47.11 | -14.44 | 34.56 | -69.11 |  | 0.84 | 1 | 0.361 |
|  | 16 |  | FC*_B_* of N_2_ & N_3_ | 8.65 | 7 |  | 11 | -46.78 | -14.11 | 34.39 | -68.78 |  | 0.51 | 1 | 0.477 |
|  | 17 |  | FC*_B_* of N_1_ & N_5_ | 8.63 | 8 |  | 11 | -47.12 | -14.45 | 34.56 | -69.12 |  | 0.84 | 1 | 0.359 |
|  | 18 |  | FC*_B_* of N_1_ & N_2_ | 8.52 | 9 |  | 11 | -46.72 | -14.05 | 34.36 | -68.72 |  | 0.44 | 1 | 0.507 |
|  | 19 |  | FC*_B_* of N_3_ & N_6_ | 8.51 | 10 |  | 11 | -46.74 | -14.07 | 34.37 | -68.74 |  | 0.46 | 1 | 0.496 |
|  | 20 |  | FC*_B_* of N_1_ & N_3_ | 8.50 | 11 |  | 11 | -46.74 | -14.07 | 34.37 | -68.74 |  | 0.46 | 1 | 0.496 |
|  | 21 |  | FC*_W_* of N_2_ | 8.49 | 12 |  | 11 | -46.80 | -14.13 | 34.40 | -68.80 |  | 0.52 | 1 | 0.470 |
|  | 22 |  | FC*_B_* of N_2_ & N_6_ | 8.49 | 13 |  | 11 | -46.58 | -13.91 | 34.29 | -68.58 |  | 0.30 | 1 | 0.581 |
|  | 23 |  | FC*_W_* of N_4_ | 8.42 | 14 |  | 11 | -46.67 | -14.00 | 34.34 | -68.67 |  | 0.40 | 1 | 0.529 |
|  | 24 |  | FC*_B_* of N_1_ & N_4_ | 8.38 | 15 |  | 11 | -46.29 | -13.62 | 34.14 | -68.29 |  | 0.01 | 1 | 0.910 |
|  | 25 |  | FC*_B_* of N_5_ & N_6_ | 8.37 | 16 |  | 11 | -46.52 | -13.85 | 34.26 | -68.52 |  | 0.24 | 1 | 0.622 |
|  | 26 |  | FC*_B_* of N_3_ & N_4_ | 8.33 | 17 |  | 11 | -46.28 | -13.61 | 34.14 | -68.28 |  | 0.01 | 1 | 0.937 |
|  | 27 |  | FC*_B_* of N_4_ & N_6_ | 8.28 | 18 |  | 11 | -46.28 | -13.61 | 34.14 | -68.28 |  | 0.00 | 1 | 0.956 |
|  | 28 |  | FC*_W_* of N_1_ | 8.28 | 19 |  | 11 | -46.28 | -13.61 | 34.14 | -68.28 |  | 0.00 | 1 | 0.975 |
|  | 29 |  | FC*_W_* of N_3_ + Task × FC*_W_* of N_3_ | 7.66 | 20 |  | 12 | -48.41 | -12.78 | 36.21 | -72.41 |  | 4.14 | 2 | 0.126 |
|  | 30 |  | FC*_W_* of N_2_ + Task × FC*_W_* of N_2_ | 6.83 | 21 |  | 12 | -46.67 | -11.04 | 35.34 | -70.67 |  | 2.40 | 2 | 0.301 |
|  | 31 |  | FC*_B_* of N_1_ & N_5_ + Task × FC*_B_* of N_1_ & N_5_ | 6.60 | 22 |  | 12 | -46.24 | -10.60 | 35.12 | -70.24 |  | 1.96 | 2 | 0.375 |
|  | 32 |  | FC*_B_* of N_2_ & N_6_ + Task × FC*_B_* of N_2_ & N_6_ | 6.55 | 23 |  | 12 | -45.66 | -10.03 | 34.83 | -69.66 |  | 1.39 | 2 | 0.500 |
|  | 33 |  | FC*_W_* of N_5_ + Task × FC*_W_* of N_5_ | 6.48 | 24 |  | 12 | -45.79 | -10.15 | 34.89 | -69.79 |  | 1.51 | 2 | 0.470 |
|  | 34 |  | FC*_B_* of N_4_ & N_5_ + Task × FC*_B_* of N_4_ & N_5_ | 6.44 | 25 |  | 12 | -45.68 | -10.04 | 34.84 | -69.68 |  | 1.40 | 2 | 0.495 |
|  | 35 |  | FC*_B_* of N_2_ & N_3_ + Task × FC*_B_* of N_2_ & N_3_ | 6.44 | 26 |  | 12 | -45.12 | -9.48 | 34.56 | -69.12 |  | 0.84 | 2 | 0.657 |
|  | 36 |  | FC*_W_* of N_4_ + Task × FC*_W_* of N_4_ | 6.25 | 27 |  | 12 | -45.51 | -9.87 | 34.75 | -69.51 |  | 1.23 | 2 | 0.540 |
|  | 37 |  | FC*_B_* of N_3_ & N_6_ + Task × FC*_B_* of N_3_ & N_6_ | 6.22 | 28 |  | 12 | -45.17 | -9.54 | 34.59 | -69.17 |  | 0.90 | 2 | 0.638 |
|  | 38 |  | FC*_B_* of N_2_ & N_5_ + Task × FC*_B_* of N_2_ & N_5_ | 6.20 | 29 |  | 12 | -45.34 | -9.70 | 34.67 | -69.34 |  | 1.06 | 2 | 0.587 |
|  | 39 |  | FC*_B_* of N_1_ & N_6_ + Task × FC*_B_* of N_1_ & N_6_ | 6.19 | 30 |  | 12 | -45.16 | -9.52 | 34.58 | -69.16 |  | 0.88 | 2 | 0.643 |
|  | 40 |  | FC*_B_* of N_1_ & N_3_ + Task × FC*_B_* of N_1_ & N_3_ | 6.18 | 31 |  | 12 | -45.15 | -9.51 | 34.57 | -69.15 |  | 0.87 | 2 | 0.646 |
|  | 41 |  | FC*_B_* of N_1_ & N_2_ + Task × FC*_B_* of N_1_ & N_2_ | 6.10 | 32 |  | 12 | -44.84 | -9.20 | 34.42 | -68.84 |  | 0.56 | 2 | 0.756 |
|  | 42 |  | FC*_B_* of N_1_ & N_4_ + Task × FC*_B_* of N_1_ & N_4_ | 6.06 | 33 |  | 12 | -44.48 | -8.84 | 34.24 | -68.48 |  | 0.20 | 2 | 0.904 |
|  | 43 |  | FC*_B_* of N_4_ & N_6_ + Task × FC*_B_* of N_4_ & N_6_ | 6.00 | 34 |  | 12 | -44.78 | -9.14 | 34.39 | -68.78 |  | 0.50 | 2 | 0.778 |
|  | 44 |  | FC*_B_* of N_3_ & N_4_ + Task × FC*_B_* of N_3_ & N_4_ | 5.86 | 35 |  | 12 | -44.29 | -8.65 | 34.14 | -68.29 |  | 0.01 | 2 | 0.995 |
|  | 45 |  | FC*_B_* of N_5_ & N_6_ + Task × FC*_B_* of N_5_ & N_6_ | 5.84 | 36 |  | 12 | -44.53 | -8.89 | 34.26 | -68.53 |  | 0.25 | 2 | 0.881 |
|  | 46 |  | FC*_W_* of N_1_ + Task × FC*_W_* of N_1_ | 5.77 | 37 |  | 12 | -44.28 | -8.64 | 34.14 | -68.28 |  | 0.00 | 2 | 0.999 |

*Notes:* FC*_W_*, within-network FC; FC*_B_*, between-network FC.

**Table S4.** Model Comparison and Selection: Swap Errors.

| Iteration | Model | Nested Model | Effect Added | REML Fit | |  | ML Fit | | | | |  | LRT Against Nested | | |
| --- | --- | --- | --- | --- | --- | --- | --- | --- | --- | --- | --- | --- | --- | --- | --- |
|  |  |  |  | LL | Order |  | *df* | AIC | BIC | LL | Deviance |  | χ^2^ | *df* | *p* |
| 1 | *Base* | - | - | 163.38 | - |  | 4 | -334.05 | -322.17 | 171.02 | -342.05 |  | - | - | - |
|  | 1 | *Base* | FC*_W_* of N_5_ | 160.62 | 1 |  | 5 | -334.68 | -319.83 | 172.34 | -344.68 |  | 2.64 | 1 | 0.105 |
|  | 2 |  | FC*_W_* of N_6_ | 160.34 | 2 |  | 5 | -334.11 | -319.27 | 172.06 | -344.11 |  | 2.07 | 1 | 0.151 |
|  | 3 |  | FC*_W_* of N_3_ | 160.32 | 3 |  | 5 | -334.06 | -319.21 | 172.03 | -344.06 |  | 2.01 | 1 | 0.157 |
|  | 4 |  | FC*_W_* of N_1_ | 160.26 | 4 |  | 5 | -333.95 | -319.10 | 171.97 | -343.95 |  | 1.90 | 1 | 0.168 |
|  | 5 |  | FC*_B_* of N_5_ & N_6_ | 160.11 | 5 |  | 5 | -333.63 | -318.78 | 171.81 | -343.63 |  | 1.58 | 1 | 0.209 |
|  | 6 |  | FC*_B_* of N_4_ & N_6_ | 159.89 | 6 |  | 5 | -333.17 | -318.32 | 171.59 | -343.17 |  | 1.12 | 1 | 0.290 |
|  | 7 |  | FC*_B_* of N_1_ & N_2_ | 159.83 | 7 |  | 5 | -333.05 | -318.20 | 171.53 | -343.05 |  | 1.00 | 1 | 0.317 |
|  | 8 |  | FC*_B_* of N_3_ & N_6_ | 159.76 | 8 |  | 5 | -332.92 | -318.07 | 171.46 | -342.92 |  | 0.87 | 1 | 0.351 |
|  | 9 |  | FC*_B_* of N_2_ & N_6_ | 159.72 | 9 |  | 5 | -332.83 | -317.98 | 171.42 | -342.83 |  | 0.78 | 1 | 0.376 |
|  | 10 |  | FC*_B_* of N_1_ & N_6_ | 159.67 | 10 |  | 5 | -332.73 | -317.88 | 171.37 | -342.73 |  | 0.68 | 1 | 0.408 |
|  | 11 |  | FC*_B_* of N_2_ & N_3_ | 159.51 | 11 |  | 5 | -332.39 | -317.54 | 171.20 | -342.39 |  | 0.34 | 1 | 0.557 |
|  | 12 |  | FC*_B_* of N_2_ & N_5_ | 159.49 | 12 |  | 5 | -332.36 | -317.51 | 171.18 | -342.36 |  | 0.31 | 1 | 0.577 |
|  | 13 |  | FC*_W_* of N_2_ | 159.48 | 13 |  | 5 | -332.33 | -317.49 | 171.17 | -342.33 |  | 0.29 | 1 | 0.593 |
|  | 14 |  | FC*_B_* of N_3_ & N_5_ | 159.47 | 14 |  | 5 | -332.32 | -317.47 | 171.16 | -342.32 |  | 0.27 | 1 | 0.604 |
|  | 15 |  | FC*_B_* of N_4_ & N_5_ | 159.40 | 15 |  | 5 | -332.18 | -317.33 | 171.09 | -342.18 |  | 0.13 | 1 | 0.716 |
|  | 16 |  | FC*_B_* of N_1_ & N_3_ | 159.40 | 16 |  | 5 | -332.17 | -317.32 | 171.09 | -342.17 |  | 0.12 | 1 | 0.725 |
|  | 17 |  | FC*_B_* of N_2_ & N_4_ | 159.38 | 17 |  | 5 | -332.14 | -317.29 | 171.07 | -342.14 |  | 0.09 | 1 | 0.768 |
|  | 18 |  | FC*_B_* of N_1_ & N_5_ | 159.38 | 18 |  | 5 | -332.12 | -317.27 | 171.06 | -342.12 |  | 0.07 | 1 | 0.784 |
|  | 19 |  | FC*_B_* of N_3_ & N_4_ | 159.36 | 19 |  | 5 | -332.09 | -317.24 | 171.04 | -342.09 |  | 0.04 | 1 | 0.840 |
|  | 20 |  | FC*_W_* of N_4_ | 159.36 | 20 |  | 5 | -332.08 | -317.23 | 171.04 | -342.08 |  | 0.03 | 1 | 0.853 |
|  | 21 |  | FC*_B_* of N_1_ & N_4_ | 159.36 | 21 |  | 5 | -332.08 | -317.23 | 171.04 | -342.08 |  | 0.03 | 1 | 0.859 |
|  | 22 |  | FC*_W_* of N_3_ + Task × FC*_W_* of N_3_ | 157.41 | 22 |  | 6 | -333.44 | -315.62 | 172.72 | -345.44 |  | 3.39 | 2 | 0.184 |
|  | 23 |  | FC*_B_* of N_2_ & N_6_ + Task × FC*_B_* of N_2_ & N_6_ | 157.09 | 23 |  | 6 | -332.77 | -314.95 | 172.39 | -344.77 |  | 2.72 | 2 | 0.256 |
|  | 24 |  | FC*_W_* of N_5_ + Task × FC*_W_* of N_5_ | 157.08 | 24 |  | 6 | -332.76 | -314.94 | 172.38 | -344.76 |  | 2.71 | 2 | 0.258 |
|  | 25 |  | FC*_W_* of N_6_ + Task × FC*_W_* of N_6_ | 156.86 | 25 |  | 6 | -332.31 | -314.49 | 172.16 | -344.31 |  | 2.26 | 2 | 0.322 |
|  | 26 |  | FC*_W_* of N_1_ + Task × FC*_W_* of N_1_ | 156.69 | 26 |  | 6 | -331.96 | -314.14 | 171.98 | -343.96 |  | 1.91 | 2 | 0.385 |
|  | 27 |  | FC*_B_* of N_4_ & N_6_ + Task × FC*_B_* of N_4_ & N_6_ | 156.66 | 27 |  | 6 | -331.90 | -314.08 | 171.95 | -343.90 |  | 1.85 | 2 | 0.397 |
|  | 28 |  | FC*_B_* of N_5_ & N_6_ + Task × FC*_B_* of N_5_ & N_6_ | 156.65 | 28 |  | 6 | -331.88 | -314.06 | 171.94 | -343.88 |  | 1.83 | 2 | 0.401 |
|  | 29 |  | FC*_B_* of N_3_ & N_6_ + Task × FC*_B_* of N_3_ & N_6_ | 156.44 | 29 |  | 6 | -331.44 | -313.62 | 171.72 | -343.44 |  | 1.39 | 2 | 0.499 |
|  | 30 |  | FC*_B_* of N_2_ & N_5_ + Task × FC*_B_* of N_2_ & N_5_ | 156.30 | 30 |  | 6 | -331.16 | -313.34 | 171.58 | -343.16 |  | 1.11 | 2 | 0.574 |
|  | 31 |  | FC*_B_* of N_1_ & N_6_ + Task × FC*_B_* of N_1_ & N_6_ | 156.30 | 31 |  | 6 | -331.15 | -313.33 | 171.57 | -343.15 |  | 1.10 | 2 | 0.577 |
|  | 32 |  | FC*_B_* of N_1_ & N_2_ + Task × FC*_B_* of N_1_ & N_2_ | 156.26 | 32 |  | 6 | -331.06 | -313.24 | 171.53 | -343.06 |  | 1.01 | 2 | 0.602 |
|  | 33 |  | FC*_B_* of N_3_ & N_5_ + Task × FC*_B_* of N_3_ & N_5_ | 156.14 | 33 |  | 6 | -330.82 | -313.00 | 171.41 | -342.82 |  | 0.77 | 2 | 0.680 |
|  | 34 |  | FC*_B_* of N_2_ & N_3_ + Task × FC*_B_* of N_2_ & N_3_ | 156.00 | 34 |  | 6 | -330.54 | -312.72 | 171.27 | -342.54 |  | 0.49 | 2 | 0.782 |
|  | 35 |  | FC*_W_* of N_2_ + Task × FC*_W_* of N_2_ | 155.92 | 35 |  | 6 | -330.38 | -312.56 | 171.19 | -342.38 |  | 0.33 | 2 | 0.847 |
|  | 36 |  | FC*_B_* of N_1_ & N_5_ + Task × FC*_B_* of N_1_ & N_5_ | 155.84 | 36 |  | 6 | -330.20 | -312.39 | 171.10 | -342.20 |  | 0.16 | 2 | 0.925 |
|  | 37 |  | FC*_B_* of N_1_ & N_3_ + Task × FC*_B_* of N_1_ & N_3_ | 155.84 | 37 |  | 6 | -330.20 | -312.38 | 171.10 | -342.20 |  | 0.15 | 2 | 0.926 |
|  | 38 |  | FC*_B_* of N_4_ & N_5_ + Task × FC*_B_* of N_4_ & N_5_ | 155.83 | 38 |  | 6 | -330.20 | -312.38 | 171.10 | -342.20 |  | 0.15 | 2 | 0.928 |
|  | 39 |  | FC*_B_* of N_2_ & N_4_ + Task × FC*_B_* of N_2_ & N_4_ | 155.81 | 39 |  | 6 | -330.16 | -312.34 | 171.08 | -342.16 |  | 0.11 | 2 | 0.947 |
|  | 40 |  | FC*_W_* of N_4_ + Task × FC*_W_* of N_4_ | 155.80 | 40 |  | 6 | -330.12 | -312.30 | 171.06 | -342.12 |  | 0.07 | 2 | 0.966 |
|  | 41 |  | FC*_B_* of N_3_ & N_4_ + Task × FC*_B_* of N_3_ & N_4_ | 155.78 | 41 |  | 6 | -330.09 | -312.27 | 171.05 | -342.09 |  | 0.05 | 2 | 0.978 |
|  | 42 |  | FC*_B_* of N_1_ & N_4_ + Task × FC*_B_* of N_1_ & N_4_ | 155.78 | 42 |  | 6 | -330.08 | -312.26 | 171.04 | -342.08 |  | 0.03 | 2 | 0.983 |

*Notes:* FC*_W_*, within-network FC; FC*_B_*, between-network FC.

**Table S5.** Model Comparison and Selection: Random Guesses.

| Iteration | Model | Nested Model | Fixed Effect Added | REML Fit | |  | ML Fit | | | | |  | LRT Against Nested | | |
| --- | --- | --- | --- | --- | --- | --- | --- | --- | --- | --- | --- | --- | --- | --- | --- |
|  |  |  |  | LL | Order |  | *df* | AIC | BIC | LL | Deviance |  | χ^2^ | *df* | *p* |
| 1 | *Base* | - | - | 52.39 | - |  | 4 | -108.94 | -97.06 | 58.47 | -116.94 |  | - | - | - |
|  | 1 | *Base* | FC*_B_* of N_3_ & N_5_ | 51.64 | 1 |  | 5 | -112.24 | -97.39 | 61.12 | -122.24 |  | 5.30 | 1 | 0.021 |
|  |  |  |  |  |  |  |  |  |  |  |  |  |  |  |  |
| 2 | 2 | *Base* + FC*_B_* of N_3_ & N_5_ | Task × FC*_B_* of N_3_ & N_5_ | 50.89 | 1 |  | 6 | -114.31 | -96.49 | 63.15 | -126.31 |  | 4.07 | 1 | 0.044 |
|  |  |  |  |  |  |  |  |  |  |  |  |  |  |  |  |
| 3 | 3 | *Base* + FC*_B_* of N_3_ & N_5_ + Task × FC*_B_* of N_3_ & N_5_ | FC*_W_* of N_6_ | 48.94 | 1 |  | 7 | -115.22 | -94.43 | 64.61 | -129.22 |  | 2.91 | 1 | 0.088 |
|  | 4 |  | FC*_B_* of N_1_ & N_5_ | 48.66 | 2 |  | 7 | -114.66 | -93.87 | 64.33 | -128.66 |  | 2.35 | 1 | 0.125 |
|  | 5 |  | FC*_B_* of N_1_ & N_6_ | 48.26 | 3 |  | 7 | -113.76 | -92.98 | 63.88 | -127.76 |  | 1.46 | 1 | 0.228 |
|  | 6 |  | FC*_B_* of N_5_ & N_6_ | 48.21 | 4 |  | 7 | -113.73 | -92.94 | 63.86 | -127.73 |  | 1.42 | 1 | 0.234 |
|  | 7 |  | FC*_B_* of N_3_ & N_6_ | 47.97 | 5 |  | 7 | -113.11 | -92.32 | 63.56 | -127.11 |  | 0.80 | 1 | 0.371 |
|  | 8 |  | FC*_B_* of N_2_ & N_5_ | 47.95 | 6 |  | 7 | -113.16 | -92.37 | 63.58 | -127.16 |  | 0.85 | 1 | 0.357 |
|  | 9 |  | FC*_B_* of N_2_ & N_6_ | 47.85 | 7 |  | 7 | -112.94 | -92.15 | 63.47 | -126.94 |  | 0.63 | 1 | 0.426 |
|  | 10 |  | FC*_B_* of N_2_ & N_4_ | 47.84 | 8 |  | 7 | -112.94 | -92.16 | 63.47 | -126.94 |  | 0.64 | 1 | 0.426 |
|  | 11 |  | FC*_B_* of N_1_ & N_2_ | 47.82 | 9 |  | 7 | -112.91 | -92.12 | 63.45 | -126.91 |  | 0.60 | 1 | 0.439 |
|  | 12 |  | FC*_B_* of N_4_ & N_5_ | 47.82 | 10 |  | 7 | -112.82 | -92.03 | 63.41 | -126.82 |  | 0.51 | 1 | 0.476 |
|  | 13 |  | FC*_B_* of N_3_ & N_4_ | 47.69 | 11 |  | 7 | -112.60 | -91.81 | 63.30 | -126.60 |  | 0.29 | 1 | 0.590 |
|  | 14 |  | FC*_W_* of N_1_ | 47.68 | 12 |  | 7 | -112.53 | -91.74 | 63.27 | -126.53 |  | 0.22 | 1 | 0.638 |
|  | 15 |  | FC*_B_* of N_2_ & N_3_ | 47.64 | 13 |  | 7 | -112.54 | -91.75 | 63.27 | -126.54 |  | 0.23 | 1 | 0.632 |
|  | 16 |  | FC*_W_* of N_5_ | 47.63 | 14 |  | 7 | -112.45 | -91.66 | 63.22 | -126.45 |  | 0.14 | 1 | 0.709 |
|  | 17 |  | FC*_B_* of N_1_ & N_4_ | 47.57 | 15 |  | 7 | -112.31 | -91.52 | 63.16 | -126.31 |  | 0.00 | 1 | 0.981 |
|  | 18 |  | FC*_B_* of N_4_ & N_6_ | 47.57 | 16 |  | 7 | -112.33 | -91.54 | 63.16 | -126.33 |  | 0.02 | 1 | 0.890 |
|  | 19 |  | FC*_W_* of N_4_ | 47.56 | 17 |  | 7 | -112.36 | -91.57 | 63.18 | -126.36 |  | 0.05 | 1 | 0.829 |
|  | 20 |  | FC*_B_* of N_1_ & N_3_ | 47.56 | 18 |  | 7 | -112.34 | -91.55 | 63.17 | -126.34 |  | 0.03 | 1 | 0.861 |
|  | 21 |  | FC*_W_* of N_3_ | 47.54 | 19 |  | 7 | -112.31 | -91.52 | 63.16 | -126.31 |  | 0.00 | 1 | 0.949 |
|  | 22 |  | FC*_W_* of N_2_ | 47.53 | 20 |  | 7 | -112.31 | -91.52 | 63.15 | -126.31 |  | 0.00 | 1 | 0.986 |
|  | 23 |  | FC*_W_* of N_6_ + Task × FC*_W_* of N_6_ | 46.50 | 21 |  | 8 | -113.84 | -90.08 | 64.92 | -129.84 |  | 3.53 | 2 | 0.171 |
|  | 24 |  | FC*_B_* of N_1_ & N_5_ + Task × FC*_B_* of N_1_ & N_5_ | 46.47 | 22 |  | 8 | -113.82 | -90.06 | 64.91 | -129.82 |  | 3.51 | 2 | 0.173 |
|  | 25 |  | FC*_B_* of N_5_ & N_6_ + Task × FC*_B_* of N_5_ & N_6_ | 45.89 | 23 |  | 8 | -112.61 | -88.86 | 64.31 | -128.61 |  | 2.31 | 2 | 0.316 |
|  | 26 |  | FC*_B_* of N_2_ & N_5_ + Task × FC*_B_* of N_2_ & N_5_ | 45.87 | 24 |  | 8 | -112.52 | -88.76 | 64.26 | -128.52 |  | 2.21 | 2 | 0.332 |
|  | 27 |  | FC*_B_* of N_1_ & N_6_ + Task × FC*_B_* of N_1_ & N_6_ | 45.79 | 25 |  | 8 | -112.28 | -88.52 | 64.14 | -128.28 |  | 1.97 | 2 | 0.373 |
|  | 28 |  | FC*_B_* of N_2_ & N_4_ + Task × FC*_B_* of N_2_ & N_4_ | 45.73 | 26 |  | 8 | -112.27 | -88.51 | 64.14 | -128.27 |  | 1.96 | 2 | 0.375 |
|  | 29 |  | FC*_B_* of N_3_ & N_6_ + Task × FC*_B_* of N_3_ & N_6_ | 45.43 | 27 |  | 8 | -111.44 | -87.68 | 63.72 | -127.44 |  | 1.13 | 2 | 0.569 |
|  | 30 |  | FC*_B_* of N_2_ & N_6_ + Task × FC*_B_* of N_2_ & N_6_ | 45.41 | 28 |  | 8 | -111.57 | -87.81 | 63.79 | -127.57 |  | 1.26 | 2 | 0.532 |
|  | 31 |  | FC*_B_* of N_1_ & N_4_ + Task × FC*_B_* of N_1_ & N_4_ | 45.31 | 29 |  | 8 | -111.27 | -87.51 | 63.64 | -127.27 |  | 0.96 | 2 | 0.619 |
|  | 32 |  | FC*_W_* of N_1_ + Task × FC*_W_* of N_1_ | 45.27 | 30 |  | 8 | -111.17 | -87.41 | 63.59 | -127.17 |  | 0.86 | 2 | 0.650 |
|  | 33 |  | FC*_B_* of N_1_ & N_2_ + Task × FC*_B_* of N_1_ & N_2_ | 45.24 | 31 |  | 8 | -111.26 | -87.50 | 63.63 | -127.26 |  | 0.95 | 2 | 0.621 |
|  | 34 |  | FC*_B_* of N_4_ & N_5_ + Task × FC*_B_* of N_4_ & N_5_ | 45.21 | 32 |  | 8 | -111.02 | -87.27 | 63.51 | -127.02 |  | 0.71 | 2 | 0.700 |
|  | 35 |  | FC*_B_* of N_2_ & N_3_ + Task × FC*_B_* of N_2_ & N_3_ | 45.17 | 33 |  | 8 | -111.11 | -87.35 | 63.55 | -127.11 |  | 0.80 | 2 | 0.671 |
|  | 36 |  | FC*_B_* of N_3_ & N_4_ + Task × FC*_B_* of N_3_ & N_4_ | 45.09 | 34 |  | 8 | -110.87 | -87.11 | 63.43 | -126.87 |  | 0.56 | 2 | 0.757 |
|  | 37 |  | FC*_B_* of N_1_ & N_3_ + Task × FC*_B_* of N_1_ & N_3_ | 45.04 | 35 |  | 8 | -110.80 | -87.05 | 63.40 | -126.80 |  | 0.50 | 2 | 0.781 |
|  | 38 |  | FC*_W_* of N_5_ + Task × FC*_W_* of N_5_ | 44.96 | 36 |  | 8 | -110.55 | -86.79 | 63.28 | -126.55 |  | 0.24 | 2 | 0.886 |
|  | 39 |  | FC*_W_* of N_4_ + Task × FC*_W_* of N_4_ | 44.86 | 37 |  | 8 | -110.45 | -86.70 | 63.23 | -126.45 |  | 0.15 | 2 | 0.929 |
|  | 40 |  | FC*_B_* of N_4_ & N_6_ + Task × FC*_B_* of N_4_ & N_6_ | 44.86 | 38 |  | 8 | -110.36 | -86.60 | 63.18 | -126.36 |  | 0.05 | 2 | 0.975 |
|  | 41 |  | FC*_W_* of N_3_ + Task × FC*_W_* of N_3_ | 44.80 | 39 |  | 8 | -110.32 | -86.56 | 63.16 | -126.32 |  | 0.01 | 2 | 0.996 |
|  | 42 |  | FC*_W_* of N_2_ + Task × FC*_W_* of N_2_ | 44.79 | 40 |  | 8 | -110.31 | -86.56 | 63.16 | -126.31 |  | 0.01 | 2 | 0.997 |

*Notes:* FC*_W_*, within-network FC; FC*_B_*, between-network FC.
